# Plant Glyoxylate/Succinic Semialdehyde Reductases: Comparative Biochemical Properties, Function during Chilling Stress, and Subcellular Localization

**DOI:** 10.3389/fpls.2017.01399

**Published:** 2017-08-14

**Authors:** Adel Zarei, Carolyne J. Brikis, Vikramjit S. Bajwa, Greta Z. Chiu, Jeffrey P. Simpson, Jennifer R. DeEll, Gale G. Bozzo, Barry J. Shelp

**Affiliations:** ^1^Department of Plant Agriculture, University of Guelph, Guelph ON, Canada; ^2^Ontario Ministry of Agriculture, Food and Rural Affairs, Simcoe ON, Canada

**Keywords:** abiotic stress, aldehyde detoxification, mutants, overexpression, redox homeostasis, substrate promiscuity

## Abstract

Plant NADPH-dependent glyoxylate/succinic semialdehyde reductases 1 and 2 (cytosolic GLYR1 and plastidial/mitochondrial GLYR2) are considered to be of particular importance under abiotic stress conditions. Here, the apple (*Malus* × *domestica* Borkh.) and rice (*Oryza sativa* L.) GLYR1s and GLYR2s were characterized and their kinetic properties were compared to those of previously characterized GLYRs from *Arabidopsis thaliana* [L.] Heynh. The purified recombinant GLYRs had an affinity for glyoxylate and succinic semialdehyde, respectively, in the low micromolar and millimolar ranges, and were inhibited by NADP^+^. Comparison of the GLYR activity in cell-free extracts from wild-type Arabidopsis and a *glyr1* knockout mutant revealed that approximately 85 and 15% of the cellular GLYR activity is cytosolic and plastidial/mitochondrial, respectively. Recovery of GLYR activity in purified mitochondria from the Arabidopsis *glyr1* mutant, free from cytosolic GLYR1 or plastidial GLYR2 contamination, provided additional support for the targeting of GLYR2 to mitochondria, as well as plastids. The growth of plantlets or roots of various Arabidopsis lines with altered GLYR activity responded differentially to succinic semialdehyde or glyoxylate under chilling conditions. Taken together, these findings highlight the potential regulation of highly conserved plant GLYRs by NADPH/NADP^+^ ratios *in planta*, and their roles in the reduction of toxic aldehydes in plants subjected to chilling stress.

## Introduction

Under stress conditions such as chilling, drought and salinity, toxic aldehydes can accumulate in plants and interact with DNA, lipids and proteins, or influence the transcription of stress-related genes, thereby causing cellular and developmental problems (Weber et al., 2004; Kotchoni et al., 2006; Yamauchi et al., 2011; Mano, 2012; Biswas and Mano, 2015; Srivastava et al., 2017; see references therein). Altering activities of enzymes that modify the aldehyde chemical grouping has been shown to affect plant tolerance to various stresses (Oberschall et al., 2000; Bouché et al., 2003; Sunkar et al., 2003; Zarei et al., 2016), indicating that detoxification of aldehydes is important for maintaining plant health.

Succinic semialdehyde (SSA) is a mitochondrially generated intermediate in the metabolism of 4-aminobutyrate (GABA), which accumulates in response to abiotic stresses (Bown and Shelp, 1997; Shelp et al., 1999; Kinnersley and Turano, 2000). SSA is typically oxidized to succinate via SSA dehydrogenase (Tuin and Shelp, 1994; Bouché et al., 2003), but evidence is available for the generation of 4-hydroxybutyrate (GHB) from SSA in response to hypoxia, high light, salinity, drought and chilling (Allan et al., 2003, 2008, 2012; Breitkreuz et al., 2003; Fait et al., 2005). Glyoxylate can be generated in various subcellular compartments and biochemical processes: the peroxisome during the photorespiratory or non-photorespiratory synthesis of serine (Bauwe et al., 2010; Hoover et al., 2013; Ros et al., 2014); the glyoxysome during fatty acid catabolism (Kunze et al., 2006); the cytosol during the interconversion of organic acids (Eprintsev et al., 2015); and the endoplasmic reticulum during purine catabolism (Werner and Witte, 2011). Glycolate, the precursor of glyoxylate in photorespiration, is known to accumulate under hypoxic conditions, and both glycolate and glyoxylate accumulate with the suppression of glycolate oxidase (Narsai et al., 2009; Lu et al., 2014).

Several mechanisms can be involved in the detoxification of glyoxylate: transamination to glycine (e.g., GABA transaminase); decarboxylation to formate; oxidation to oxalate; and, reduction to glycolate (Givan and Kleczkowski, 1992; Bauwe et al., 2010; Peterhansel et al., 2010; Shelp et al., 2012a; Igamberdiev and Eprintsev, 2016; see references therein). Our laboratory has biochemically characterized two NADPH-dependent glyoxylate reductases (EC 1.1.1.79) from *Arabidopsis thaliana* (L.) Heynh. (GLYR1, GenBank accession no. AAK94781; GLYR2, (GenBank accession no. AAP42747) that efficiently convert glyoxylate into glycolate (Hoover et al., 2007a,b; Simpson et al., 2008). Notably, they also convert SSA into GHB, albeit with lower catalytic efficiency than for the glyoxylate to glycolate conversion (2–12 vs. 660–2870 s^-1^ mM^-1^, see **Table 1** for additional information), and constitutive expression of *AtGLYR1* in an SSA dehydrogenase-deficient yeast enables growth on 20 μM GABA and significantly enhances cellular GHB levels (Breitkreuz et al., 2003). Furthermore, *glyr1* and *glyr2* single mutants of Arabidopsis accumulate less GHB with submergence stress than the wild-type (WT) (Allan et al., 2012). A *gaba transaminase*/*ssa dehydrogenase* double mutant of Arabidopsis accumulates both SSA and GHB, and is more sensitive to exogenous SSA or GHB than WT plants (Ludewig et al., 2008). Both WT and *glyr1/2* double mutants of Arabidopsis are more sensitive to exogenous SSA than GHB under ambient conditions, suggesting that GHB accumulation in *ssa dehydrogenase* mutants and during abiotic stress is a response for avoiding SSA toxicity (Mekonnen and Ludewig, 2016). Further research is required to establish whether a differential phenotype is associated with the *glyr1/glyr2* double mutants under abiotic stress conditions.

**Table 1:** Comparison of the kinetic parameters for purified recombinant GLYRs from apple, rice, and Arabidopsis.

Varied	Fixed	*K*_m_	*V*_max_	*k*_cat_	*k*_cat_/*K*_m_
substrate	substrate	μM	μmol min^-1^	*s*^-1^	*s*^-1^ mM^-1^
			mg^-1^ protein		
*Md*GLYR1					
Glyoxylate	NADPH	14.1 ± 2.1	54.7 ± 6.7	30.9 ± 3.8	2230 ± 153
NADPH	Glyoxylate	3.3 ± 0.5	39.4 ± 6.1	22.3 ± 3.4	6803 ± 278
SSA	NADPH	1133 ± 273	8.1 ± 2.9	4.6 ± 1.6	5.1 ± 2.3
NADPH	SSA	7.4 ± 1.9	9.8 ± 1.0	5.5 ± 0.6	832 ± 166
*Md*GLYR2					
Glyoxylate	NADPH	23.9 ± 1.9	34.3 ± 3.7	19.1 ± 2.1	796 ± 33
NADPH	Glyoxylate	1.8 ± 0.5	28.2 ± 2.7	15.7 ± 1.5	10070 ± 2275
SSA	NADPH	6500 ± 327	18.6 ± 3.4	10.3 ± 1.9	1.6 ± 0.2
NADPH	SSA	11.7 ± 3.5	15.5 ± 1.5	8.6 ± 0.9	833 ± 183
*Os*GLYR1					
Glyoxylate	NADPH	53.2 ± 11.1	65.8 ± 16.5	33.7 ± 7.5	636 ± 99
NADPH	Glyoxylate	8.8 ± 2.5	28.5 ± 5.9	14.2 ± 2.9	1729 ± 201
SSA	NADPH	4003 ± 341	43.9 ± 8.5	21.9 ± 4.2	5.4 ± 0.9
NADPH	SSA	36.2 ± 2.1	21.4 ± 5.0	10.7 ± 2.5	290 ± 51
*Os*GLYR2					
Glyoxylate	NADPH	19.1 ± 9	18.6 ± 7.9	10.8 ± 4.6	349 ± 16
NADPH	Glyoxylate	8.4 ± 1.2	15.3 ± 2.9	8.9 ± 1.7	1040 ± 73
SSA	NADPH	1457 ± 124	12.4 ± 3.5	7.2 ± 2.0	5.1 ± 1.8
NADPH	SSA	12.5 ± 0.6	11.3 ± 0.3	6.5 ± 0.2	525 ± 40
*At*GLYR1					
Glyoxylate	NADPH	23.2 ± 3.2	56.7 ± 5.6	28.4 ± 2.8	1259 ± 188
Glyoxylate^1^	NADPH	4.5 ± 0.9	15.2 ± 2.3	13.1 ± 1.9	2870 ± 453
NADPH^1^	Glyoxylate	2.2 ± 0.3	21.6 ± 2.4	9.3 ± 0.1	4370 ± 446
SSA^1^	NADPH	870 ± 40	23.5 ± 1.5	10.1 ± 0.4	11.6 ± 2.2
NADPH^1^	SSA	2.6 ± 0.1	18.8 ± 3.3	8.1 ± 0.0	3500 ± 208
*At*GLYR2					
Glyoxylate	NADPH	19.3 ± 4.0	36.8 ± 19.9	18.4 ± 10.0	906 ± 353
Glyoxylate^2^	NADPH	34.2 ± 3.3	40.6 ± 8.7	22.5 ± 4.8	660 ± 130
NADPH^2^	Glyoxylate	1.4 ± 0.2	23.0 ± 2.2	12.0 ± 0.8	8550 ± 960
SSA^2^	NADPH	8960 ± 710	30.7 ± 4.0	17.0 ± 2.2	1.9 ± 0.3
NADPH^2^	SSA	1.2 ± 0.2	19.3 ± 1.5	10.7 ± 0.8	9180 ± 1620

*Data from this study were collected using a Spectramax Plus384 Absorbance Microplate Reader and represent the mean *±* SE of two (Arabidopsis*)* or three (apple and rice) enzyme preparations. The *V*_*max*_ values obtained with glyoxylate and NADPH or SSA and NADPH were not influenced by whether the substrate or co-factor was varied or fixed, as indicated by a *t*-test with *P* = 0.05 as the significance threshold. Previously published data were collected using a Cary 300 double-beam absorption spectrophotometer (^1^Hoover et al., 2007a; ^2^Simpson et al., 2008) and represent the mean *±* SE of three enzyme preparations.*

Arabidopsis GLYR1 and GLYR2 activities have strong affinity for NADPH (*K*_m_ = 1.2–2.6 μM; see **Table 1** for additional information), regardless of whether glyoxylate or SSA is the substrate, and *At*GLYR1 is known to be competitively inhibited by NADP^+^ (Hoover et al., 2007a,b; Simpson et al., 2008). The ratio of NADPH/NADP^+^ in mature leaves of Arabidopsis and tobacco increases from approximately 2 to 3–5 with submergence and heat stresses, and to 8 with chilling (Allan et al., 2008). In the absence of applied stress, the NADPH/NADP^+^ ratio in an *NAD KINASE1* overexpression line of Arabidopsis is increased by threefold, and there is a corresponding increase in GHB accumulation (Allan et al., 2012).

Arabidopsis *GLYR1* and *GLYR2* are moderately expressed throughout the plant, including roots and imbibed seed, but *GLYR2* expression is more highly associated than *GLYR1* with rosette leaves, which are known to highly express photorespiratory genes (Foyer et al., 2009; Shelp et al., 2012b). With the exception of two studies (Allan et al., 2008, 2012), there is little evidence for the stress-induced expression of GLYRs (Shelp et al., 2012b; Zarei et al., 2017; see references therein). Furthermore, transient GHB accumulation in cold-acclimated Arabidopsis plants (Kaplan et al., 2007) and rice seed germinated under hypoxia (Narsai et al., 2009) seems to be independent of *GLYR* expression (see Shelp et al., 2012b). Overall, these *in vitro* and *in planta* studies support the operation and redox regulation of glyoxylate- and SSA-dependent GLYR activities under abiotic stress conditions, as suggested previously (Allan et al., 2008, 2012).

Early research indicated that NADPH-dependent GLYR activity is located in both cytosol and plastids (Givan et al., 1988; also see review by Givan and Kleczkowski, 1992). Recently, Simpson et al. (2008), Ching et al. (2012), Brikis et al. (2017) established, using various transient and stable expression systems, in combination with appropriate organelle markers, that GLYR1s from apple (*Malus* × *domestica* Borkh.) and rice (*Oryza sativa* L.), as well as Arabidopsis, are exclusively cytosolic, whereas the GLYR2s are localized to both mitochondria and plastids. Apple and rice GLYRs have not yet been biochemically characterized.

In the present study, we demonstrated that: (i) GLYRs from apple and rice, like those from Arabidopsis, display higher affinity and catalytic efficiency for glyoxylate than for SSA; (ii) the activity of apple GLYRs is inhibited by NADP^+^; (iii) the growth of plantlets or roots of various Arabidopsis lines with altered GLYR activity respond differentially to exogenous SSA, GHB or glyoxylate under chilling conditions; and (iv) approximately 85 and 15% of the total GLYR activity in Arabidopsis is present in the cytosol and plastids/mitochondria, respectively. Taken together, these findings highlight the potential roles of GLYRs in the reduction of toxic aldehydes within distinct compartments of the plant cell during exposure to chilling conditions.

## Materials and Methods

### Plant Material, and Gene Expression and Southern Analysis

*Arabidopsis thaliana* (L.) Heynh. ecotype Columbia (Col-0) was the genetic background for the WT and all transgenic/mutant lines. Seeds were sterilized in a closed container using a 3–4 h exposure to vaporized chlorine gas^1^. Sterilized seeds were typically grown as a lawn on Petri plates containing half-strength Murashige and Skoog medium (Murashige and Skoog, 1962) with 0.7% agar and appropriate antibiotic for selection of transgenic plants. Following stratification of the seeds for 3 days at 4°C, the plates were transferred to a controlled environment chamber (Enconair, Winnipeg, Canada) and supplied with a 16-h light (Sylvania Model 3500K) period at 60 μmol s^-1^ m^-2^ at 23°C and an 8-h dark period at 21°C.

Total RNA was extracted from 100 to 1000 mg of liquid N_2_-frozen Arabidopsis rosette leaves (Zarei et al., 2011), rice (*O. sativa* L.) leaves (Brauer et al., 2011) and mature apple (*Malus* × *domestica* Borkh.) fruit (Gasic et al., 2004). RNA was treated with DNase I using the TURBO DNA-free kit (Applied Biosystems, Austin, TX, United States) according to the manufacturer’s manual. One microgram total RNA was used for first-strand cDNA synthesis with Oligo(dT)20 and Superscript III (Invitrogen, Carlsbad, CA, United States) at 50°C according to the manufacturer’s protocol. Primers used for quantitative real-time PCR (qPCR) analyses were designed using Primer Express 3 software (Applied Biosystem, Austin TX, United States). Primers with 90–105% efficiency were selected for further analysis.

Quantitative PCR was performed according to standard methods with an iQ5 Multicolor Real-Time PCR Detection system (Bio-Rad Laboratories). The primer sequences used to determine the abundance of GLYR1, GLYR2 and housekeeping transcripts are listed in Supplementary Table S1 (RTAtGLYR1-F/R, RTAtGLYR2-F/R, RTAtEF-1-F/R, and 18S rRNA- F/R). The housekeeping gene *ELONGATION FACTOR-1*α (*EF-1*α, At5g60390) was chosen based on the research by Czechowski et al. (2005). Relative expression and data analysis were determined using the 2^-ΔCt^ method (Livak and Schmittgen, 2001). Two technical replicates were conducted for each biological replicate, and the mean ± SE of three biological replicates was determined for each genotype.

The presence and copy number of transgene inserts in GLYR1 overexpression (Ox) lines were determined by Southern hybridization as described by McLean et al. (2003). Genomic DNA was isolated as described by Montiel et al. (2011). About 5 μg of genomic DNA was digested with *Nco*I, separated on a 1% (w/v) agarose gel, and transferred to a positively charged nylon membrane (Roche Applied Science, Laval, QC, Canada). The membrane was probed, processed, and labeled bands were detected according to digoxigenin (DIG)-labeling kit protocols (Roche Applied Science). The probe was generated by PCR from the cDNA using Probe-GLYR1-F and Probe-GLYR1-R primers (Supplementary Table S1) in the presence of DIG-deoxy-uridine triphosphate. The forward primer flanked an intron/exon boundary in the *GLYR1* gene to prevent GLYR1 genomic DNA amplification during probe synthesis. Hybridization was performed with a DIG-labeled probe according to the most stringent hybridization protocols of the manufacturer (Roche Applied Science). The membrane was exposed under a Fluorchem 8800 Imaging System (Alpha Innotech Inc., Miami, FL, United States) for 16 min to visualize labeled bands.

### Cloning of cDNAs Encoding Apple and Rice GLYRs and Arabidopsis GLYR2

The predicted amino acid sequences of Arabidopsis GLYR1 (*At*3g25530, GenBank Acc. No. NM_113449) and GLYR2 (*At*1g17650, GenBank Acc. no. NM_101628) were obtained from The Arabidopsis Information Resource^2^. A truncated *AtGLYR2* lacking its N-terminal 58 amino acid-long predicted plastid targeting sequence (*AtGLYR2*Δ*58*) was amplified from the Arabidopsis cDNA using primers VB-F4 and VB-R4 and cloned into pET15b using *Nde*I and *BamH*I restriction enzymes to yield pET15b-*AtGLYR2*Δ*58* construct. All primer sequences used to clone the various genes are listed in Supplementary Table S1. When expressed, the truncated *At*GLYR2 possesses an N-terminal 6×His tag for purification. The *AtGLYR1* expression construct was made elsewhere (Hoover et al., 2007b).

Two apple *GLYRs* were identified in the apple genome database^3^. Two products (958- and 1166-bp), designated as *MdGLYR1* (GenBank Acc No. KT202799) and *MdGLYR2* (GenBank Acc No. KT202800), respectively, were amplified from apple fruit cDNA cv. Empire and appropriate primers (CT-F12 and CT-R12 for the *MdGLYR1* gene, and CT-F13 and CT-R13 for the *MdGLYR2* gene). The resulting open reading fragments (ORF) were cloned into pCR2.1-TOPO (Invitrogen). The predicted translation products are 58% identical to each other. *Md*GLYR1 is 80% identical to *At*GLYR1, and *Md*GLYR2 is 78% identical to *At*GLYR2. The primers CT-F17 and CT-R17 were used to amplify *MdGLYR1* with 5′ *Nde*I and 3′ *BamH*I restriction sites. TargetP^4^ (v1.1; Emanuelsson et al., 2000) predicted that *MdGLYR2* has an N-terminal 52 amino acid plastid targeting sequence. The primers CT-F20 and CT-R20 were used to amplify the *MdGLYR2* ORF minus the sequence encoding the putative N-terminal plastid transit peptide with 5′ *Nde*I and 3′ *BamH*I restriction sites. Both sets of PCR products were digested with *Nde*I and *BamH*I and ligated into pET15b expression vector (Novagen, Cambridge, MA, United States) to produce pET15b-*MdGLYR1* and pET15b-*MdGLYR2*Δ*54*. When expressed, the truncated *Md*GLYR2 possesses an N-terminal 6×His tag.

The *AtGLYR1* and *AtGLYR2* cDNAs were blasted against the rice genome annotation project^5^ to reveal two full-length cDNA sequences, LOC_*Os*02g35500 and LOC_*Os*01g39270, which are 81 and 65% identical to *At*GLYR1 and *At*GLYR2, respectively. These sequences were designated as putative *Os*GLYR1 and *Os*GLYR2, respectively. Full-length *OsGLYR1* and *OsGLYR2* were amplified from the rice cv. Kaybonnet cDNA using forward and reverse primers, VB-F1 and VB-R1 for *OsGLYR1*, and VB-F2 and VB-R2 for *OsGLYR2*. TargetP (v1.1^4^; Emanuelsson et al., 2000) predicted that *Os*GLYR2 contains an N-terminal 35 amino acid-long plastid targeting sequence. The primers VB-F3 and VB-R2 were used to amplify *OsGLYR2*, minus the sequence encoding the protein’s putative plastid transit peptide. The last two sets of PCR products were cloned into pET15b with *NdeI* and *BamHI* enzymes to yield pET15b-*OsGLYR1* and pET15b-*OsGLYR2*Δ*35*. When expressed, the truncated *Os*GLYRs possess an N-terminal 6×His tag for purification. All *GLYR* genes PCR-amplified from cDNAs were sequenced after cloning.

### Production, Purification and Kinetic Analysis of Recombinant Apple, Rice and Arabidopsis GLYRs

The constructs pET15b-*MdGLYR1* and pET15b-*MdGLYR2*Δ*54* were individually expressed in *Escherichia coli* BL-21(DE3) Rosetta (pLysS) cells (Novagen) co-expressing the GroES/GroEL chaperone complex, and the resulting soluble recombinant proteins were subjected to affinity chromatography on a Ni^2+^ column essentially as described previously (Clark et al., 2009). Also, the pET15b-*AtGLYR1*, pET15b-*AtGLYR2*Δ*58*, pET15b-*OsGLYR1*, and pET15b-*OsGLYR2*Δ*35* constructs were expressed in *E. coli* BL-21(DE3) cells (Novagen) and the recombinant proteins purified as described in Hoover et al. (2007a). Each eluted protein was precipitated with 80% (w/v) ammonium sulfate, stored at -80°C, and the pellet was resuspended, as necessary, in 100 μL of reaction buffer [50 mM 4-(2-hydroxyethyl)-1-piperazineethanesulfonic acid (HEPES), 10% sorbitol (w/v), pH 7.8 for *At*GLYR1 and *At*GLYR2, pH 7.5 and 7.3 for *Md*GLYR2 and *Md*GLYR2, and pH 6.5 and 7.1 for *Os*GLYR1 and *Os*GLYR2Δ35, respectively].

Enzymatic activity was monitored as the oxidation of NAD(P)H at room temperature using a Spectramax Plus384 Absorbance Microplate Reader (Molecular Devices, Sunnyvale, CA, United States). The 250-μL assay was initiated with a pre-made master mix of buffer, co-factor and substrate. Reaction rates were proportional to the enzyme concentrations used in the assays (2–50 nM). The optimal pH for enzymatic activity at 50 μM NADPH and 50 μM glyoxylate was determined using buffers (50 mM each) with overlapping pH range [2-morpholino-ethanesulfonic acid (MES) for pH 5.5–6.8; HEPES for pH 6.8–8.2; and *N*-tris(hydroxymethyl)methyl-4-aminobutanesulfonic acid (TABS) for pH 8.2–9.6]. Initially, the three buffers were tested separately for the apple isoforms, then they were combined for rice. The influence of redox ratio on the activities of *Md*GLYR1 and *Md*GLYR2 was investigated using 50 μM glyoxylate, 50 μM NADPH and 0–0.5 mM NADP^+^. The kinetic parameters (*V*_max_, *K*_m_, *k*_cat_, *k*_cat_/*K*_m_) for the purified enzymes were determined using a mixture of the MES, HEPES, and TABS (50 mM each) at the pH optimum for the enzyme source under consideration, with saturating co-factor (50 μM NADPH) and varying substrate (up to 200 μM glyoxylate or 10 mM SSA) or varying co-factor (up to 100 μM NADPH) and saturating substrate (50 μM glyoxylate or 3.75–10 mM SSA) as described previously (Hoover et al., 2007a). For each enzyme source the concentration of substrate or co-factor was varied to give five to six data points both above and below the *K*_m_. The dependence of enzymatic activity on pH or redox ratio was determined as the mean ± SD of three to four technical measurements of a typical enzyme preparation (i.e., one biological replicate), whereas the kinetic parameters were expressed as the mean ± SE of three biological replicates, each measured using four technical replicates.

### Generation of Arabidopsis Lines with Altered *GLYR* Expression

Arabidopsis *glyr1* and *glyr2* single mutants (Salk_057410 and GK316D04, respectively) were twice backcrossed to the WT. Plants homozygous for *glyr1* T-DNA were identified using the glyr1-RP and glyr1-LP primers for the WT allele, and LBb1.3 and glyr1-RP for T-DNA insertion (as recommended by the Salk Institute). The primers were designed using the Salk iSECT tool online software. Similarly, homozygous *glyr2* was identified using glyr2-RP and glyr2-LP primers for the WT allele, and GABI-Kat T-DNA border primer and glyr2-RP for the T-DNA. The double *glyr1*/*glyr2* mutant was generated by crossing healthy plants of the homozygous *glyr1* and *glyr2* single mutants. With the exception of three to five closed flowers with white petals on the main shoot of the mother plant, all flowers and siliques were removed using scissors and forceps. The intact flowers on the female parent were emasculated with the aid of a dissecting microscope. Open flowers with yellow anthers were removed from the male parent and brushed against the stigmatic surface of carpels on the female parent. Female plants were labeled and covered with porous plastic bags until fruit maturity (approximately 4 weeks later). Screening of the T_2_ population was based on the gene-specific and T-DNA primers given above.

Arabidopsis *glyr1*RNAi and *glyr2* RNAi lines were generated in *glyr2* T-DNA and *glyr1* T-DNA mutant backgrounds, respectively. To prepare hairpin RNA constructs, a 233-bp fragment (from +722 to +955 relative to the transcriptional initiation site) for *AtGLYR1* and a 382-bp fragment (from +630 to +1012) for *AtGLYR2* were each amplified by PCR from cDNA with the following primer pairs containing a LB clonase site: Clonase-GLYR1-F/Clonase-GLYR1-R and Clonase-GLYR2-F/Clonase-GLYR2-R, respectively. These amplified fragments were separately cloned into the intermediate clonase vector pDONR221, followed by assembling the fragments into a gateway binary RNAi vector pB7GWIWG2 (II) by the LR clonase site reaction^6^ (Karimi et al., 2002). The binary vectors *GLYR1*-pB7GWIWG2(II) and *GLYR2*-pB7GWIWG2(II) were introduced into *Agrobacterium tumefaciens* strain EHA105 according to Hood et al. (1993). The *GLYR1* and *GLYR2* RNAi constructs were introduced, respectively, into the Arabidopsis *glyr2* and *glyr1* T-DNA mutant backgrounds using the floral dip method (Clough and Bent, 1998). Seeds were selected on solid half-strength MS medium containing 20 mg L^-1^ Basta (as a T-DNA selection marker) and 100 mg L^-1^ timentin (to prevent *Agrobacterium* growth), and then 14-days-old plants were transferred to Sunshine LC1 potting mix (Sun Gro Horticulture, Canada) and placed in a controlled environment growth chamber (Enconair Model GC8-2H set at 60 μmol s^-1^ m^-2^ photosynthetic photon flux density at 23°C for 16 h and no light at 21°C for 8 h), where they were allowed to self-pollinate and mature. T_2_ progenies were again selected on half-strength MS medium containing Basta and then transferred to LC1 potting mix. Thirty seedlings were separately screened at the rosette stage for the *GLYR1* and *GLYR2* genes using qPCR (Supplementary Figure S1); the primer sets are given in Supplementary Table S1. Seeds of selected lines were collected for further experiments.

For generation of *AtGLYR1* overexpression (Ox) lines, the full-length cDNA sequence for *AtGLYR1* was amplified using *Spe*I-*GLYR1*-F and *Spe*I-*GLYR1*-R primers and cloned into a prepared pMdM7 binary vector (Ni et al., 1995) and transformed into *A. tumefaciens* strain LB4404. Plant transformation, transformant screening, tissue culture and growth conditions were similar to above, except that seed were selected on medium containing 50 mg L^-1^ kanamycin. The three transgenic lines that were chosen for further analysis had two to three times the *GLYR1* transcript level of the WT (Supplementary Figure S2). Assays of GLYR activity in desalted cell-free leaf extracts with 50 μM glyoxylate and NADPH (Simpson et al., 2008) revealed that two of the three lines had 70–90% higher activity than the WT. Southern blot analysis revealed weak signals, if at all, in the WT lane, perhaps as a results of only partial binding between the unprocessed endogenous gene and the probe. However, it was clear that the two lines with highest GLYR activity carried two to three copies, of the transgene. The line with the highest activity and lowest transgene copy number was selected for further experimentation.

### Chilling Phenotype of Arabidopsis Lines with Altered *GLYR* Expression

In preliminary chilling trials, five sterilized seeds from the Arabidopsis WT and each of the *GLYR1* Ox, *glyr1/2* and *glyr1/2*-RNAi lines were sown along the center of 14-cm Petri plates containing half-strength MS medium with 0.6% (w/v) agar, 30 g L^-1^ sucrose, 1 mM MES (pH 5.8) and SSA or GHB (0–3 mM) and then subjected to a stratification regime (3 days, 4°C, dark). Thereafter, the plates were stored vertically in a tissue culture chamber under 12-h period at 22°C for 12 days or 10°C for 27 days (photosynthetic flux density of 60 μmol m^-2^ s^-1^). The experimental design was completely randomized and comprised of three plate replicates for each treatment.

In follow up trials, sterilized seeds from the WT and *glyr1/2* and *GLYR1* Ox lines were sown as a lawn on 150-mm Petri plates containing half-strength MS medium with 0.6% (w/v) agar, 30 g L^-1^ sucrose, and 1 mM MES (pH 5.8), subjected to a stratification regime (3 days, 4°C, dark), and then placed in a tissue culture chamber (22°C, 14 h light/18°C, 10 h dark, photosynthetic flux density of 120 μmol m^-2^ s^-1^) for 8 days. Seedlings from all three genotypes were placed along the center of individual 14-cm plates containing the same medium supplemented with glyoxylate (0–2 mM) or SSA (0–3 mM) and the primary root apex was noted on the exterior surface of the plate. Then the plates were stored vertically at 10°C for another 8 days and photographed. The gain in root growth was estimated using ImageJ software^7^. The experimental design was completely randomized and comprised of 10 plate replicates for each treatment.

### Distribution of GLYR Activity in Cytosolic, Plastidial, and Mitochondrial Fractions from Arabidopsis

Crude homogenates of rosette leaf tissue from 4-week-old Arabidopsis WT or *glyr1* mutant (grown as described above), were prepared using the extraction medium [0.3 M sucrose, 25 mM Na_4_P_2_O_7_, 2 mM sodium ethylenediaminetetraacetate, 10 mM KH_2_PO_4_, 1% (w/v) polyvinylpyrrolidone-40, 1% bovine serum albumin] described by Taylor et al. (2014). Then the homogenates were sonicated for 10 s using a sonic dismembrator (Fisher Scientific, Model 120), and pelleted by centrifugation. The supernatants were desalted using Econo-Pac 10DG columns (Bio-Rad) and assayed for GLYR activity with 50 μM glyoxylate and NADPH. Intact mitochondria were also isolated from 50 g of homogenized rosette tissue of the *glyr1* mutant grown under a 23°C 11-h light (120 μmol m^-2^ s^-1^)/19°C 13-h dark regime according to Taylor et al. (2014). Briefly, the crude homogenate was differentially centrifuged to give an organelle suspension, which was then applied to a linear polyvinylpyrrolidone-40 gradient (0–4.4% v/v) in 28% (w/v) Percoll and centrifuged at 40,000 × *g* for 40 min. This enabled recovery of a white/pale brown mitochondrial band toward the bottom of the gradient (separated from plastid thylakoid and peroxisomal fractions), which was then concentrated and washed repeatedly using differential centrifugation. Aliquots of the organelle suspension and purified mitochondria fractions were sonicated, pelleted by centrifugation, and desalted as described above. GLYR activity was assayed with 50 μM glyoxylate and NADPH, and mitochondrial fumarase and plastid phosphoribulokinase were assayed as described previously (Taylor et al., 2014), with the exception that the reaction volume was 250 μL and the wavelength used for the fumarase assay was 240 nm (Puchegger et al., 1990), rather than 340 nm as given.

## Results

### Characterization of Recombinant Apple, Rice, and Arabidopsis GLYRs

**FIGURE 1:**
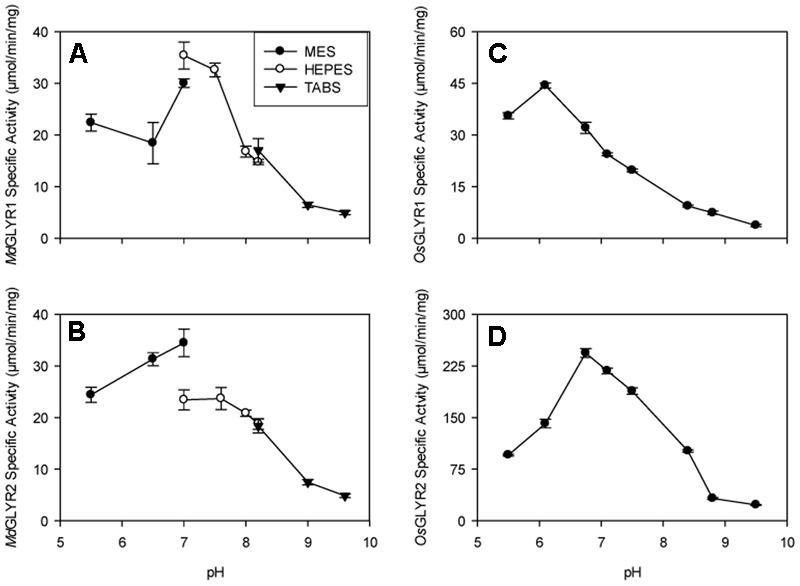
Dependence of recombinant apple and rice GLYR activities on pH. The left and right panels, represent apple **(A,B)** and rice **(C,D)** GLYRs, respectively, whereas the upper and lower panels represent GLYR1s **(A,C)** and GLYR2s **(B,D)**, respectively. The data, expressed as specific activity (μmol min^-1^ mg^-1^ protein), were determined using 50 μM glyoxylate and NADPH in thee buffers (50 mM each): 2-morpholino-ethanesulfonic acid, 4-(2-hydroxyethyl)-1-piperazineethanesulfonic acid and *N*-tris(hydroxymethyl)methyl-4-aminobutanesulfonic acid either separately **(A,B)** or together **(C,D)**. Each datum represents the mean (±SD) of three technical measurements of a typical enzyme preparation.

The predicted N-terminal targeting presequences in GLYR2s from apple, rice, and Arabidopsis (Brikis et al., 2017) were removed and the resulting truncated sequences (i.e., *Md*GLYR2Δ54, *Os*GLYR2Δ35, and *At*GLYR2Δ58), as well as the corresponding full-length GLYR1s from the same three plant species (i.e., *Md*GLYR1, *Os*GLYR1, and *At*GLYR1), were individually expressed in *E. coli*. This allowed for the recovery of a significant portion of the recombinant GLYR2 proteins, similar to the GLYR1 proteins, in the corresponding soluble fraction (Supplementary Figure S3). In all fractions eluted from Ni^2+^-affinity columns, the recombinant protein was purified to near homogeneity. Predicted protein masses for *Md*GLYR1, *Md*GLYR2Δ54, *Os*GLYR1, *Os*GLYR2Δ35, *At*GLYR1, and *At*GLYR2Δ58 were 31.8, 33.1, 30.5, 31.4, 30.7, and 33.2 kDa, respectively. The mass of recombinant *At*GLYR2Δ58 appeared slightly larger than predicted. The scientific literature contains many examples of cytosolic proteins that migrate during SDS polyacrylamide electrophoresis at rates that are inconsistent with their molecular mass (see Shi et al., 2012). While such “gel shifting” could be caused by single amino acid substitutions, we repeatedly cloned and sequenced the gene and prepared the recombinant *At*GLYR2Δ58 protein with similar results. It is possible that interaction between SDS and the mutant protein is modified by the presence of the N-terminal 6×His motif. The activities of *Md*GLYR1and *Os*GLYR1 were maximal at pH 6.8 and 6, respectively, although both enzymes retained at least half of maximal activity over the pH range of 5.5–7. The maximal activities of *Md*GLYR2 and OsGLYR2 were found to be at approximately pH 6.8 and at least 50% of maximal activity was retained in the pH range of 6–8 (**Figure 1**).

**FIGURE 2:**
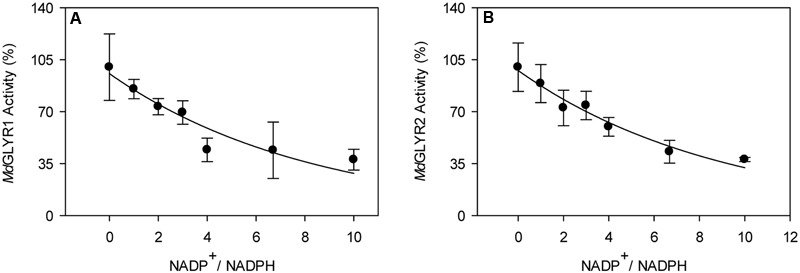
Dependence of recombinant apple GLYR activities on NADP^+^/NADPH ratio. The activities of *Md*GLYR1 **(A)** and *Md*GLYR2Δ54 **(B)** were determined using 50 μM glyoxylate and NADPH, and increasing concentrations of NADP^+^. Data represent the mean ± SD of four technical measurements of a typical enzyme preparation.

In the present study, enzymatic activity for the various recombinant proteins was determined using a microplate reader. A preliminary study of glyoxylate-dependent activity in rice GLYR1 revealed that 50 and 100 μM NADPH gave approximately 4.5 times the activity with corresponding levels of NADH (data not shown); therefore, detailed NADH kinetics for both rice and apple GLYRs were not determined here. The kinetic data for the apple and rice proteins with NADPH as the co-factor represent the mean (±SE) of three replicate preparations, and the *V*_max_ values obtained with glyoxylate or SSA were not significantly influenced by whether the substrate or co-factor was varied or fixed (*P* > 0.05, *t*-test) (**Table 1**). Glyoxylate- dependent kinetics with NADPH were also determined here for the *At*GLYR1 and the *At*GLYR2 using the microplate reader, so that direct comparisons could be made with published values obtained with the use of a Cary 300 spectrophotometer. Overall, the catalytic efficiencies (*k*_cat_/*K*_m_) of the apple and rice GLYRs, like those for the Arabidopsis GLYRs (Hoover et al., 2007a; Simpson et al., 2008), were within the same order of magnitude for each substrate, and the *K_m_* values for glyoxylate and NADPH were in the micromolar range, whereas the *K_m_* values for SSA were in the millimolar range (see **Table 1** and Supplementary Figures S4–S6). For all GLYRs, the highest catalytic efficiencies were observed for NADPH, mostly at fixed glyoxylate concentrations, followed by glyoxylate at fixed NADPH concentrations, whereas catalytic efficiencies for SSA were 0.2 to 1.4% of those for glyoxylate (**Table 1**). Overall, the catalytic efficiencies of GLYR1 and GLYR2 for glyoxylate or SSA in the NADPH-dependent reaction ranged approximately three- to four-fold only across the species compared here, and there were no identifiable catalytic differences between the GLYR1 and GLYR2 isoforms, indicating that all GLYRs prefer glyoxylate over SSA, and have a high affinity for their co-substrate NADPH. The activities of *Md*GLYR1 and *Md*GLYR2 displayed inhibitory responses to an increasing NADP^+^/NADPH ratio (**Figure 2**), and their reaction rates were reduced by half with an NADP^+^/NADPH ratio of 5.

### Phenotypic Response to Chilling of Various GLYR Lines Supplied with Exogenous Glyoxylate, Succinic Semialdehyde, or γ-Hydroxybutyrate

**FIGURE 3:**
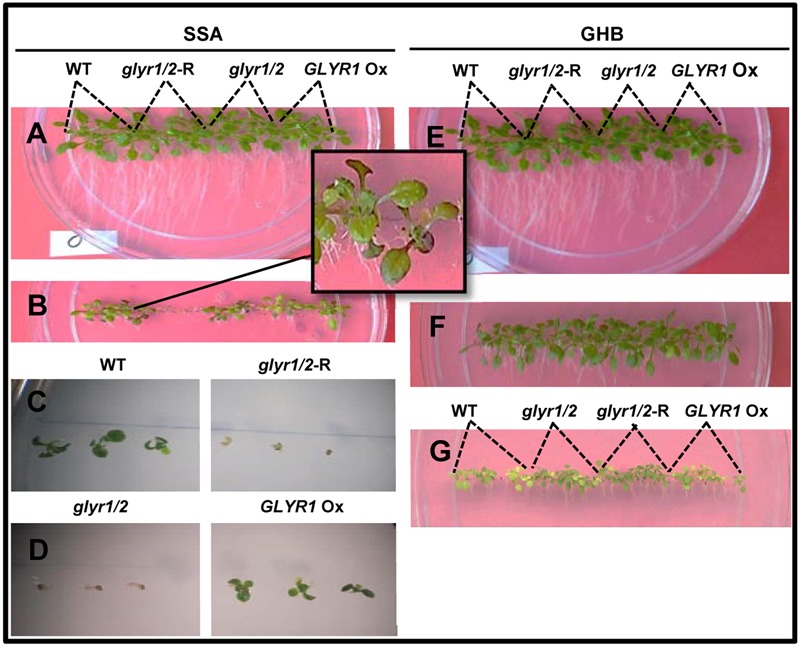
Impact of Arabidopsis genotype on the early phenotypic response to succinic semialdehyde or γ-hydroxybutyrate under chilling stress. Seeds of wild-type (WT) plants and *glyr1/2-*RNAi, *glyr1/2*, and *GLYR1* Ox were sown on plates containing half-strength MS medium with up to 3 mM SSA or GHB and grown at 10°C for 27 days. Levels of SSA or GHB are 0 **(A,E)**, 1 **(B)**, 2 **(C,D,F),** and 3 **(G)** mM.

To investigate whether GLYR activity is linked to a phenoptypic response to chilling, seeds of Arabidopsis WT, *GLYR1* Ox, *glyr1/2* and *glyr1/2*-RNAi were sown directly on agar medium and grown at 22°C or 10°C in separate tissue culture chambers. At 22°C, the early growth of the different genotypes was similar in the absence or presence of added SSA or GHB up to 12 days (data not shown). The early growth of the genotypes at 10°C was also similar in the absence of glyoxylate or GHB after 27 days (**Figures 3A,E**). However, when SSA or GHB (up to 3 mM each) was added to the medium, a differential response was observed among the genotypes at 10°C. That is, increasing concentrations of SSA progressively inhibited growth and development (**Figures 3B–D**). At 1 mM SSA, the inhibition was greater with *glyr1/2-*RNAi and *glyr1/2* than the WT and *GLYR1* Ox, and purple tinging of the leaves was repeatedly observed in the WT (**Figure 3B**). At 2 mM SSA, radicle development in both *glyr1/2-*RNAi and *glyr1/2* was halted, and shoot production was entirely absent (**Figures 3C,D**). Higher magnification observations from a replicate experiment indicated that 2 mM SSA stunted radicle growth in all four lines, and confirmed that shoot and leaf development was hindered in *glyr1/2-*RNAi and *glyr1/2* (**Figures 3C,D**). In general, the plantlets were more tolerant to GHB than SSA. Root length was reduced with 2 and 3 mM GHB, and shoot reduction and yellowing were particularly evident at 3 mM GHB; notably, the WT and *GLYR1* Ox line were more sensitive to GHB than *glyr1/2* and *glyr1/2*-RNAi (**Figures 3E–G**).

**FIGURE 4:**
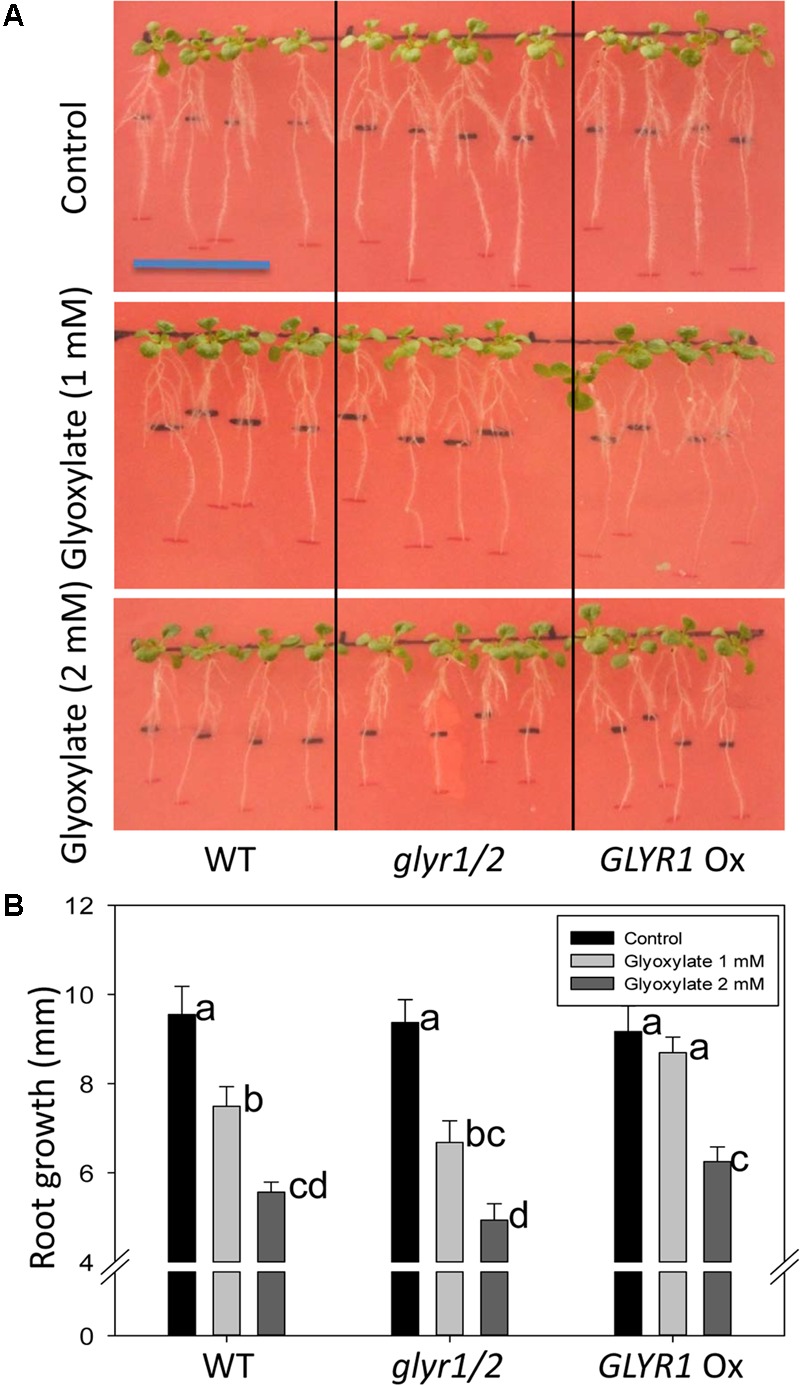
Impact of Arabidopsis genotype on the response of root growth to glyoxylate under chilling stress. Seeds of WT, *glyr1/2* and *GLYR1* Ox were sown on plates containing half-strength MS medium and grown at ambient temperature for 8 days. Then the plantlets were transferred to new agar plates containing 0 (control), 1 or 2 mM glyoxylate, as indicated on the y-axis, grown at 10°C for another 8 days, and then the gain in root length was determined **(A)**. Results are the least squared means ± SE of measurements from 10 plates (three plants per plate) estimated following two-way analysis of variance (ANOVA) performed in PROC MIXED procedure in SAS with *P* = 0.05 as the significance threshold. **(B)** Error bars sharing the same letter are not significantly different.

To further investigate whether GLYR activity could be linked to a phenotypic response, Arabidopsis WT, and transgenic *GLYR1* Ox and *glyr1/2* plantlets were transferred to a chilling temperature of 10°C for an 8-days period in the presence of glyoxylate or SSA. In the absence of glyoxylate, all genotypes displayed a similar gain in root length; however, the addition of glyoxylate generally decreased this gain (**Figure 4A**). Notably, the *GLYR1* Ox line gained more root length than WT and *glyr1/2* mutant plants with 1 mM glyoxylate, whereas *GLYR1* Ox gained more root length than *glyr1/2* but not the WT in the presence of 2 mM glyoxylate (**Figure 4B**). We were unable to demonstrate the same differential response to SSA using this experimental design (data not shown).

### Subcellular Localization of GLYR Activity in Arabidopsis

**FIGURE 5:**
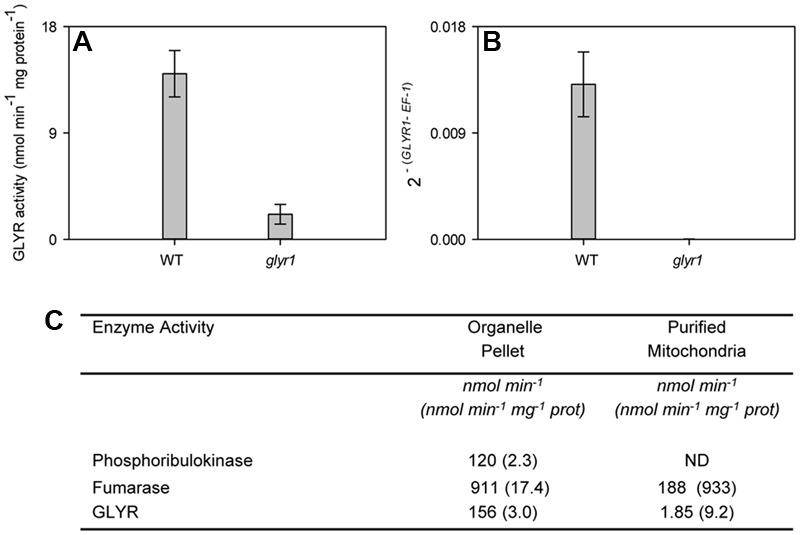
Mitochondrial localization of GLYR2 activity in rosette leaf tissue from Arabidopsis. Total GLYR activity **(A)** and *GLYR1* expression **(B)** in WT and *glyr1* knockout mutant. Data represent the mean ± SE of three biological replicates. GLYR activity (total per fraction, or protein bases) in the organelle suspension and purified mitochondria from *glyr1* mutant **(C)**. Data represent a preparation of 50 g of rosette leaf tissue; ND, not detected. Similar results were obtained with a replicate preparation.

As expected, *AtGLYR1* expression in leaves of the Arabidopsis *glyr1* mutant was not detectable, and the total GLYR activity in crude tissue homogenates was markedly less than that in the WT (**Figures 5A,B**), indicating that cytosolic *At*GLYR1 and plastidial/mitochondrial *At*GLYR2 could be responsible for approximately 85 and 15% of the total GLYR activity, respectively, in leaves. To further assess whether *At*GLYR2 is located in mitochondria, as well as plastids, mitochondria were purified from a leaf organelle suspension from the *glyr1* mutant. The organelle suspension and purified mitochondria were assayed for marker enzymes and GLYR activity. As shown in **Figure 5C**, the activities of plastidial phosphoribulokinase, mitochondrial fumarase and GLYR were present in the organelle suspension. Phosphoribulokinase activity was not detectable in the purified mitochondria, confirming that this fraction was essentially free of plastid contamination and, hence, plastidial *At*GLYR2. The specific activity of fumarase was enriched approximately 53-fold in the purified mitochondria, compared to the organelle suspension, whereas the specific activity of GLYR was enriched approximately 2.1-fold. These findings lend further support for the notion that *At*GLYR2 is indeed localized in mitochondria, as well as plastids.

## Discussion

The recombinant apple and rice GLYR1s and GLYR2s investigated here were highly active with glyoxylate and NADPH over a relatively broad pH range from slightly acidic to slightly basic, regardless of whether the different pH buffers were combined or tested separately in the assay mixtures (**Figure 1**). These results are in agreement with previous studies of the recombinant Arabidopsis GLYRs (Hoover et al., 2007b; Simpson et al., 2008), and the purified spinach enzyme (Kleczkowski et al., 1986). Our kinetic analyses showed that recombinant GLYRs from the monocotyledonous rice, as well as the dicotyledonous apple and Arabidopsis, behaved similarly with respect to their catalytic efficiencies (**Table 1**), probably because of their conserved active site residues and NADPH-binding sequence (Hoover et al., 2013; Brikis et al., 2017). The *K*_m_ values for glyoxylate and SSA were 5–53 μM and 0.8–9 mM, respectively, with NADPH as the co-factor (**Table 1**), whereas the *K*_m_ for glyoxylate was 60 μM for the purified spinach enzyme (Kleczkowski, 1995). Thus, it can be concluded that all plant GLYRs have a preference for glyoxylate over SSA. There are few reliable data on the pool sizes of glyoxylate and SSA in plants, perhaps in part because of their propensity to form adducts (Nikiforova et al., 2014; also see references therein). The level of glyoxylate in rice seedlings is 0.27 μmol g^-1^ fresh mass (FM) (Yu et al., 2010) or 0.27 mM (assuming 1 g FM equals 1 mL H_2_O), which is within the micromolar range known to inhibit photosynthesis in intact chloroplasts (Campbell and Ogren, 1990). The product of glyoxylate reduction, glycolate, is known to accumulate in rice seedlings germinated under hypoxic conditions (Narsai et al., 2009). SSA levels are low (10–16 μM) in various parts of Arabidopsis plants (Palanivelu et al., 2003; Toyokura et al., 2011). However, accumulation of GHB, the product of SSA reduction, increases in Arabidopsis and tobacco plants from approximately 0.04 μmol g^-1^ FM to 0.12–2.4 μmol g^-1^ FM with the imposition of salinity, submergence, heat and chilling stresses (Allan et al., 2008), and submergence-induced GHB accumulation is markedly reduced in Arabidopsis *glyr1* and *glyr2* mutants (Allan et al., 2012).

The *K*_m_s for NADPH were 2–9 μM with the recombinant apple, rice, and Arabidopsis GLYRs (**Table 1**), and 3–6 μM for the purified spinach enzyme (Kleczkowski et al., 1986; Kleczkowski, 1995). NADPH was preferred over NADH as the co-factor for both *Os*GLYR1 and *At*GLYR1 (see Results; Hoover et al., 2007a), results in general agreement with those for the purified spinach enzyme (note: *K*_m_s for glyoxylate and NADH are 0.3 and 1.1 mM, respectively, in combination with a lower estimated *V*_max,_ Kleczkowski, 1995). Our previous results indicated that the *K*_i_ for NADP^+^, a competitive inhibitor of the glyoxylate- and SSA-dependent reactions, is 3.1–9.5 μM for *At*GLYR1 (Hoover et al., 2007b). This is much lower than the corresponding value (*K*_i_ = 60 μM) for the purified spinach enzyme (Kleczkowski, 1995), and seemingly at odds with the slight inhibition of apple GLYRs by NADP^+^ (i.e., approximately 50% inhibition with a fivefold increase in NADP^+^, **Figure 2**). The estimated ratios of NADPH:NADP^+^ in intact leaves of Arabidopsis (Allan et al., 2008, 2012), cytosol of spinach, barley, and pea leaves (Heineke et al., 1991; Wigge et al., 1993; Kleczkowski, 1995; Igamberdiev and Gardeström, 2003), and spinach chloroplasts (Heineke et al., 1991) are approximately 2, 1–4, and 0.5, respectively. Thus, it can be concluded that all plant GLYRs have a high affinity for NADPH, and NADP^+^ is not likely to be an important regulator of GLYR activity under ambient conditions, as suggested earlier (Kleczkowski, 1995). Notably, the estimated NADPH/NADP^+^ ratios in Arabidopsis and tobacco leaves have been shown to increase by 0.5- to 4-fold with submergence, heat and chilling stresses (Allan et al., 2008). In the absence of applied stress, the NADPH/NADP^+^ ratio in an *NAD KINASE1* Ox line of Arabidopsis is increased by threefold, and there is a corresponding increase in GHB accumulation (Allan et al., 2012).

Taken together, these findings are in agreement with the suggestion that GLYR activities are stimulated by stress, likely in response to the elevated supply of one or both substrates, glyoxylate and SSA, as well as the co-factor NADPH (Hoover et al., 2007a; Allan et al., 2008, 2012), despite the low affinity of GLYRs for SSA and the low tissue levels of SSA under ambient conditions. Mekonnen and Ludewig (2016) have shown the growth of both *glyr1/2* double mutants and WT plants is more sensitive to exogenous SSA than GHB at ambient temperature. Since chilling is known to cause the accumulation of both GHB and its precursor GABA in Arabidopsis within hours of exposure, and greatly enhance the leaf NADPH:NADP^+^ ratio (Allan et al., 2008), we predicted that chilling would generate a differential phenotype among the GLYR genotypes. Indeed, plantlets of the various genotypes did display a qualitative difference in the early growth response when grown on increasing levels of exogenous SSA or GHB at 10°C, but not 22°C (**Figure 3**). It was also possible to quantitatively demonstrate that the root growth of established plantlets of the various genotypes responded differentially to exogenous glyoxylate (but not SSA) with chilling (**Figure 4**). Since roots are unlikely to generate photorespiratory glyoxylate, these findings suggest that GLYRs function in the reduction of toxic aldehydes, including glyoxylate from multiple metabolic pathways (e.g., glyoxylate from non-photorespiratory serine synthesis, fatty acid catabolism, interconversion of organic acids, purine catabolism (see Introduction for citations), in plants subjected to chilling stress.

The reduction of glyoxylate can also be catalyzed by various hydroxypyruvate reductases (Kleczkowski et al., 1991; Timm et al., 2011). For example, in Arabidopsis, there are three isoforms: a peroxisomal HPR1 with a preference for NADH and hydroxypyruvate over NADPH and glyoxylate, respectively; a cytosolic HPR2 with a preference for NADPH over NADH, but similar preference for hydroxypyruvate and glyoxylate; and, a plastidial HPR3 with a preference for NADPH and glyoxylate over NADH and hydroxypyruvate, respectively (Timm et al., 2011). Our research has used transient and stable expression systems to unambiguously demonstrate that apple, rice, and Arabidopsis GLYR1s are localized to the cytosol, whereas the corresponding GLYR2s are localized in both plastids and mitochondria (Ching et al., 2012; Brikis et al., 2017). Here, approximately 85% of the total glyoxylate-reducing activity in Arabidopsis leaf extracts was lacking in the *glyr1* mutant (**Figure 5**), indicating that GLYR1, rather than HPR2, is responsible for the majority of the cytosolic activity. Furthermore, the recovery of glyoxylate-reducing activity in purified mitochondria from the *glyr1* mutant, free from cytosolic GLYR1, plastidial GLYR2 (**Figure 5**) or plastidial HPR3 (Timm et al., 2011) contamination, provided *in planta* support for the dual targeting of GLYR2. The percentage distribution of GLYR2 between mitochondria and plastids remains uncertain, although oxalate may be a useful tool to distinguish between plastidial GLYR2 and HPR3 activities (Kleczkowski et al., 1991). Early studies, using the photosynthesizing protist *Euglena gracilis*, reported the presence of NADPH-dependent GLYR activity in the intermembrane space of mitochondria, which constitutes approximately 2% of total mitochondrial protein (Yokota and Kitaoka, 1979; Yokota et al., 1985). The *K*_m_ values for NADPH and glyoxylate for this enzyme are in the low micromolar range and the pH optimum is 6.45 (Yokota et al., 1985), which are similar to our findings with GLYRs from Arabidopsis, apple and rice (**Table 1**).

In summary, the ability of GLYRs to promiscuously catalyze the reduction of SSA, as well as glyoxylate, appears to have physiological implications for the plant response to chilling conditions and could be relevant to other abiotic stresses. The generation of SSA is generally restricted to mitochondria with the catabolism of GABA (Shelp and Zarei, 2017; see references therein), whereas glyoxylate can be derived from multiple biochemical processes and subcellular compartments. With abiotic stresses, the detoxification of SSA could be diverted from the generation of succinate to GHB as SSA dehydrogenase activity becomes restricted with the accumulation of NADH (António et al., 2016). With the exception of hypoxia, abiotic stresses generally cause stomatal closure and result in corresponding increases in Rubisco oxygenase activity (Allan et al., 2009). This could increase the production and levels of glyoxylate, so that its detoxification could require capacity in addition to the photorespiration-associated transaminase activities (i.e., serine/asparagine:glyoxylate and glutamate:glyoxylate transaminases) (Peterhansel et al., 2010; Modde et al., 2017): reduction to glycolate (GLYR and HPR); decarboxylation to formate, and, oxidation to oxalate (Igamberdiev and Eprintsev, 2016; see references therein). On the other hand, with hypoxia glycolate oxidase and glycine decarboxylase activities could be inhibited (Allan et al., 2009), and detoxification of non-photorespiratory sources of glyoxylate could occur via a combination of reduction (GLYR and HPR), GABA transamination, and decarboxylation and oxidation activities.

## Author Contributions

BS conceived and supervised the project. AZ, CB, VB, and GC conducted the experiments and data analysis. JS prepared and identified the Arabidopsis *GLYR1* overexpression lines. JD and GB discussed the project. AZ, CB, GB, and BS wrote and/or edited the manuscript. All authors read and approved the final manuscript.

## Conflict of Interest Statement

The authors declare that the research was conducted in the absence of any commercial or financial relationships that could be construed as a potential conflict of interest.

## Footnotes

1 http://plantpath.wisc.edu/çafb/vapster.html

2 https://www.arabidopsis.org/index.jsp

3 www.rosaceae.org

4 http://www.cbs.dtu.dk/services/TargetP/

5 http://rice.plantbiology.msu.edu/

6 http://www.psb.ugent.be/gateway/

7 http://rsbweb.nih.gov/ij/

## References

[B1] Allan W. L. Breitkreuz K. E. Waller J. C. Simpson J. P. Hoover G. J. Rochon A. (2012). Detoxification of succinic semialdehyde in Arabidopsis glyoxylate reductase and NAD kinase mutants subjected to submergence stress. *Botany* 90 51–61. 10.1139/b11-083

[B2] Allan W. L. Clark S. M. Hoover G. J. Shelp B. J. (2009). Role of plant glyoxylate reductases during stress: a hypothesis. *Biochem. J.* 423 15–22. 10.1042/BJ20090826 19740079 PMC2762691

[B3] Allan W. L. Peiris C. Bown A. W. Shelp B. J. (2003). Gamma-hydroxybutyrate accumulates in green tea and soybean sprouts in response to oxygen deficiency. *Can. J. Plant Sci.* 83 951–953. 10.4141/P03-085

[B4] Allan W. L. Simpson J. P. Clark S. M. Shelp B. J. (2008). γ-Hydroxybutyrate accumulation in Arabidopsis and tobacco plants is a general response to abiotic stress: putative regulation by redox balance and glyoxylate reductase isoforms. *J. Exp. Bot.* 59 2555–2564. 10.1093/jxb/ern122 18495640 PMC2423657

[B5] António C. Päpke C. Rocha M. Diab H. Limami A. M. Obata T. (2016). Regulation of primary metabolism in response to low oxygen availability as revealed by carbon and nitrogen isotope redistribution. *Plant Physiol.* 170 43–56. 10.1104/pp.15.00266 26553649 PMC4704563

[B6] Bauwe H. Hagemann M. Fernie A. R. (2010). Photorespiration: players, partners and origin. *Trends Plant Sci.* 15 330–336. 10.1016/j.tplants.2010.03.006 20403720

[B7] Biswas M. D. Mano J. (2015). Lipid peroxide-derived short-chain carbonyls mediate hydrogen peroxide-induced and salt-induced programmed cell death in plants. *Plant Physiol.* 168 885–898. 10.1104/pp.115.256834 26025050 PMC4741343

[B8] Bouché N. Fait A. Bouchez D. Moller G. G. Fromm H. (2003). Mitochondrial succinic-semialdehyde dehydrogenase of the γ-aminobutyrate shunt is required to restrict levels of reactive oxygen intermediates in plants. *Proc. Natl. Acad. Sci. U.S.A.* 100 6843–6848. 10.1073/pnas.1037532100 12740438 PMC164534

[B9] Bown A. W. Shelp B. J. (1997). The metabolism and functions of gamma-aminobutyric acid. *Plant Physiol.* 115 1–5. 10.1104/pp.115.1.1 12223787 PMC158453

[B10] Brauer E. K. Rochon A. Bi Y. Bozzo G. G. Rothstein S. J. Shelp B. J. (2011). Reappraisal of nitrogen use efficiency in rice overexpressing *glutamine synthetase1*. *Physiol. Plant.* 141 361–372. 10.1111/j.1399-3054.2011.01443.x 21214879

[B11] Breitkreuz K. E. Allan W. L. Van Cauwenberghe O. R. Jakobs C. Talibi D. André B. (2003). A novel γ-hydroxybutyrate dehydrogenase: identification and expression of an Arabidopsis cDNA and potential role under oxygen deficiency. *J. Biol. Chem.* 278 41552–41556. 10.1074/jbc.M305717200 12882961

[B12] Brikis C. J. Zarei A. Trobacher C. P. DeEll J. R. Akama K. Mullen R. T. (2017). Ancient plant glyoxylate/succinic semialdehyde reductases: GLYR1s are cytosolic, whereas GLYR2s are localized to both mitochondria and plastids. *Front. Plant Sci.* 8:601 10.3389/fpls.2017.00601 PMC5399074 28484477

[B13] Campbell W. J. Ogren W. L. (1990). Gloxylate inhibition of ribulosebisphosphate carboxylase/oxygenase activation in intact, lysed, and reconstituted chloroplasts. *Photosynth. Res.* 23 257–268. 10.1007/BF00034856 24419649

[B14] Ching S. L. K. Gidda S. K. Rochon A. Van Cauwenberghe O. R. Shelp B. J. Mullen R. T. (2012). Glyoxylate reductase isoform 1 is localized in the cytosol and not peroxisomes in plant cells. *J. Integr. Plant Biol.* 54 152–168. 10.1111/j.1744-7909.2012.01103.x 22309191

[B15] Clark S. M. Di Leo R. Dhanoa P. K. Van Cauwenberghe O. R. Mullen R. T. Shelp B. J. (2009). Biochemical characterization, mitochondrial localization, expression, and potential functions for an Arabidopsis γ-aminobutyrate transaminase that utilizes both pyruvate and glyoxylate. *J. Exp. Bot.* 60 1743–1757. 10.1093/jxb/erp044 19264755 PMC2671622

[B16] Clough S. J. Bent A. F. (1998). Floral dip: A simplified method for Agrobacterium-mediated transformation of *Arabidopsis thaliana*. *Plant J.* 16 735–743. 10.1046/j.1365-313x.1998.00343.x 10069079

[B17] Czechowski T. Stitt M. Altmann T. Udvardi M. K. (2005). Genome-wide identification and testing of superior reference genes for transcript normalization. *Plant Physiol.* 139 5–17. 10.1104/pp.105.063743 16166256 PMC1203353

[B18] Emanuelsson O. Nielsen H. Brunak S. von Heijne G. (2000). Predicting subcellular localization of proteins based on their N-terminal amino acid sequence. *J. Mol. Biol.* 300 1005–1016. 10.1006/jmbi.2000.3903 10891285

[B19] Eprintsev A. T. Feedorin D. N. Salnikov A. V. Igamberdiev A. U. (2015). Expression and properties of the glyoxysomal and cytosolic forms of isocitrate lyase in *Amaranthus caudatus* L. *J. Plant Physiol.* 181 1–8. 10.1016/j.jplph.2015.02.014 25955696

[B20] Fait A. Yellin A. Fromm H. (2005). GABA shunt deficiencies and accumulation of reactive oxygen intermediates: insight from Arabidopsis mutants. *FEBS Lett.* 579 415–420. 10.1016/j.febslet.2004.12.004 15642352

[B21] Foyer C. H. Bloom A. J. Queval G. Noctor G. (2009). Photorespiratory metabolism genes, mutants, energetics, and redox signaling. *Annu. Rev. Plant Biol.* 60 455–484. 10.1146/annurev.arplant.043008.091948 19575589

[B22] Gasic K. Hernandez A. Korban S. S. (2004). RNA extraction from different apple tissues rich in polyphenols and polysaccharides for cDNA library construction. *Plant Mol. Biol. Rep.* 22 437–438. 10.1007/BF02772687

[B23] Givan C. V. Kleczkowski L. A. (1992). The enzymic reduction of glyoxylate and hydroxypyruvate in leaves of higher plants. *Plant Physiol.* 100 552–556. 10.1104/pp.100.2.552 16653027 PMC1075593

[B24] Givan C. V. Tsutakawa S. Hodgson J. M. David N. Randall D. D. (1988). Glyoxylate reductase activity in pea leaf protoplasts. Nucleotide specificity and subcellular location. *J. Plant Physiol.* 132 593–599. 10.1016/S0176-1617(88)80260-8

[B25] Heineke D. Riens B. Grosse H. Hoferichter P. Peter U. Flügge U.-I. (1991). Redox transfer across the inner chloroplast envelope membrane. *Plant Physiol* 95 1131–1137. 10.1104/pp.95.4.1131 16668101 PMC1077662

[B26] Hood E. E. Gelvin S. B. Melchers L. S. Hoekema A. (1993). New *Agrobacterium* helper plasmids for gene transfer to plants. *Transgenic Res.* 2 208–218. 10.1007/BF01977351

[B27] Hoover G. J. Jørgensen R. Rochon A. Bajwa V. S. Merrill A. R. Shelp B. J. (2013). Identification of catalytically important amino acid residues for enzymatic reduction of glyoxylate in plants. *Biochim. Biophys. Acta* 1834 2663–2671. 10.1016/j.bbapap.2013.09.013 24076009

[B28] Hoover G. J. Prentice G. A. Merrill A. R. Shelp B. J. (2007a). Glyoxylate reductase: studies of initial velocity, dead-end inhibition and product inhibition. *Can. J. Bot.* 85 896–902. 10.1139/B07-082

[B29] Hoover G. J. Van Cauwenberghe O. R. Breitkreuz K. E. Clark S. M. Merrill A. R. Shelp B. J. (2007b). Glyoxylate reductase: general biochemical properties and substrate specificity for the recombinant protein, and developmental expression and implications for glyoxylate and succinic semialdehyde metabolism in planta. *Can. J. Bot.* 85 883–895. 10.1139/B07-081

[B30] Igamberdiev A. U. Eprintsev A. T. (2016). Organic acids: the pools of fixed carbon involved in redox regulation and energy balance in higher plants. *Front. Plant Sci.* 7:1042 10.3389/fpls.2016.01042 PMC4945632 27471516

[B31] Igamberdiev A. U. Gardeström P. (2003). Regulation of NAD and NADP-dependent isocitrate dehydrogenases by reduction of levels of pyridine nucleotides in mitochondria and cytosol of pea leaves. *Biochim. Biophys. Acta* 1606 117–125. 10.1016/S0005-2728(03)00106-3 14507432

[B32] Kaplan F. Kopka J. Sung D. Y. Zhao W. Popp M. Porat M. (2007). Transcript and metabolite profiling during cold acclimation of Arabidopsis reveals an intricate relationship of cold-regulated gene expression with modifications in metabolite content. *Plant J.* 50 967–981. 10.1111/j.1365-313X.2007.03100.x 17461790

[B33] Karimi M. Inze D. Depicker A. (2002). GATEWAY vectors for Agrobacterium-mediated plant transformation. *Trends Plant Sci.* 7 193–195. 10.1016/S1360-1385(02)02251-3 11992820

[B34] Kinnersley A. M. Turano F. J. (2000). γ-Aminobutyric acid (GABA) and plant responses to stress. *CRC Crit. Rev. Plant Sci.* 19 479–509. 10.1016/S0735-2689(01)80006-X

[B35] Kleczkowski L. A. (1995). Kinetics and regulation of the NAD(P)H-dependent glyoxylate-specific reductase from spinach leaves. *Z. Naturforsch.* 50 21–28.

[B37] Kleczkowski L. A. Randall D. D. Blevins D. G. (1986). Purification and characterization of a novel NADPH(NADH)-dependent glyoxylate reductase from spinach leaves. *Biochem. J* 239 653–659. 10.1042/bj2390653 3548703 PMC1147336

[B38] Kleczkowski L. A. Randall D. D. Edwards G. E. (1991). Oxalate as a potent and selective inhibitor of spinach (*Spinacia oleracea*) leaf NADPH-dependent hydroxypyruvate reductase. *Biochem. J.* 276 125–127. 10.1042/bj2760125 2039466 PMC1151153

[B39] Kotchoni S. O. Kuhns C. Ditzer A. Kirch H. H. Bartels D. (2006). Overexpression of different aldehyde dehydrogenase genes in *Arabidopsis thaliana* confers tolerance to abiotic stress and protects plants against lipid peroxidation and oxidative stress. *Plant Cell Environ.* 29 1033–1048. 10.1111/j.1365-3040.2005.01458.x 17080931

[B40] Kunze M. Pracaroenwattana I. Smith S. M. Hartig A. (2006). A central role for the peroxisomal membrane in glyoxylate cycle function. *Biochim. Biophys. Acta* 1763 1441–1452. 10.1016/j.bbamcr.2006.09.009 17055076

[B41] Livak K. J. Schmittgen T. D. (2001). Analysis of relative gene expression data using real-time quantitative PCR and the 2^-ΔCt^ method. *Methods* 25 402–408. 10.1006/meth.2001.1262 11846609

[B42] Lu Y. Li Y. Yang Q. Zhang Z. Chen Y. Zhang S. (2014). Suppression of glycolate oxidase causes glyoxylate accumulation that inhibits photosynthesis through deactivating Rubisco in rice. *Physiol. Plant.* 150 463–476. 10.1111/ppl.12104 24102419

[B43] Ludewig F. Hüser A. Fromm H. Beauclair L. Bouché N. (2008). Mutants of GABA transaminase (POP2) suppress the severe phenotype of *succinic semialdehyde dehydrogenase* (*ssadh*) mutants in Arabidopsis. *PLoS ONE* 3:e3383 10.1371/journal.pone.0003383 PMC2557145 18846220

[B44] Mano J. (2012). Reactive carbony species: their production from lipid peroxides, action in environmental stress, and the detoxification mechanism. *Plant Physiol. Biochem.* 59 90–97. 10.1016/j.plaphy.2012.03.010 22578669

[B45] McLean M. D. Yevtushenko D. P. Deschene A. Van Cauwenberghe O. R. Makhmoudova A. Potter J. W. (2003). Overexpression of glutamate decarboxylase in transgenic tobacco plants confers resistance to the northern root-knot nematode. *Mol. Breed.* 11 277–285. 10.1023/A:1023483106582

[B46] Mekonnen D. W. Ludewig F. (2016). Phenotypic and chemotypic studies using Arabidopsis and yeast reveal that GHB coverts to SSA and induces toxicity. *Plant. Mol. Biol.* 91 429–440. 10.1007/s11103-016-0475-6 27037708

[B47] Modde K. Timm S. Florian A. Michl K. Fernie A. R. Bauwe H. (2017). High serine:glyoxylate aminotransferase activity lowers leaf daytime serine levels, inducing the phosphoserine pathway in Arabidopsis. *J. Exp. Bot.* 68 643–656. 10.1093/jxb/erw467 28011718 PMC5441925

[B48] Montiel G. Zarei A. Körbes A. P. Memelink J. (2011). The jasmonate-responsive element from the ORCA3 promoter from *Catharanthus roseus* is active in Arabidopsis and is controlled by the transcription factor *AtMYC2*. *Plant Cell Physiol.* 52 578–587. 10.1093/pcp/pcr016 21306988

[B49] Murashige T. Skoog F. (1962). A revised medium for rapid growth and bioassays with tobacco tissue cultures. *Physiol. Plant.* 15 473–497. 10.1111/j.1399-3054.1962.tb08052.x

[B50] Narsai R. Howell K. A. Carroll A. Ivanova A. Millar A. H. Whelan J. (2009). Defining core metabolic and transcriptomic responses to oxygen availability in rice embryos and young seedlings. *Plant Physiol.* 151 306–322. 10.1104/pp.109.142026 19571305 PMC2736006

[B51] Ni M. Cul D. Einstein J. Narasimhulu S. Vergara C. E. Gelvin S. B. (1995). Strength and tissue specificity of chimeric promoters derived from the octopine and manopine synthase genes. *Plant J.* 7 661–676. 10.1046/j.1365-313X.1995.7040661.x

[B52] Nikiforova V. J. Giesbertz P. Wiemer J. Bethan B. Looser R. Liebenberg V. (2014). Gloxylate, a new marker metabolite of Type 2 diabetes. *J. Diabetes Res.* 2014:685204 10.1155/2014/685204 PMC4265698 25525609

[B53] Oberschall A. Deak M. Torok K. Sass L. Vass I. Kovacs I. (2000). A novel aldose/aldehyde reductase protects transgenic plants against lipid peroxidation under chemical and drought stresses. *Plant J.* 24 437–446. 10.1111/j.1365-313X.2000.00885.x 11115125

[B54] Palanivelu R. Brass L. Edlund A. F. Preuss D. (2003). Pollen tube growth and guidance is regulated by POP2, an Arabidopsis gene that controls GABA levels. *Cell* 114 47–59. 10.1016/S0092-8674(03)00479-3 12859897

[B55] Peterhansel C. Horst I. Niessen M. Blume C. Kebish R. Kürkcüoglu S. (2010). *Photorespiration. The Arabidopsis Book.* Rockville, MD: The American Society of Plant Biologists. 10.1199/tab.0130 PMC3244903 22303256

[B56] Puchegger S. Redl B. Stöffler G. (1990). Purification and properties of a thermostable fumarate hydratase from the archaeobacterium *Sulfolobus solfataricus*. *J. Gen. Microbiol.* 136 1537–1541. 10.1099/00221287-136-8-1537 2124611

[B57] Ros R. Muñoz-Bertomeu J. Krueger S. (2014). Serine in plants: biosynthesis, metabolism, and functions. *Trends Plant Sci.* 19 564–569. 10.1016/j.tplants.2014.06.003 24999240

[B58] Shelp B. J. Bown A. W. McLean M. D. (1999). Metabolism and functions of gamma-aminobutyric acid. *Trends Plant Sci.* 4 446–452. 10.1016/S1360-1385(99)01486-7 10529826

[B59] Shelp B. J. Bozzo G. G. Trobacher C. P. Brikis C. J. Chiu G. Bajwa V. S. (2012a). Strategies and tools for studying GABA metabolism and function: I. Pathway structure. *Botany* 90 651–668. 10.1139/B2012-030

[B60] Shelp B. J. Bozzo G. G. Zarei A. Simpson J. P. Trobacher C. P. Allan W. L. (2012b). Strategies and tools for studying the metabolism and function of γ-aminobutyrate in plants. II. Integrated analysis. *Botany* 90 781–793. 10.1139/B2012-041

[B61] Shelp B. J. Zarei A. (2017). Subcellular compartmentation of 4-aminobutyrate (GABA) metabolism in Arabidopsis: an update. *Plant Signal. Behav.* 12:e13222244 10.1080/15592324.2017.1322244 PMC5501244 28448196

[B62] Shi Y. Mowery R. A. Ashley J. Hentz M. Ramirez A. J. Bilgicer B. (2012). Abnormal SDS-PAGE migration of cytosolic proteins can identify domains and mechanisms that control surfactant binding. *Protein Sci.* 21 1197–1209. 10.1002/pro.2107 22692797 PMC3537240

[B63] Simpson J. P. Di Leo R. Dhanoa P. K. Allan W. L. Makhmoudova A. Clark S. M. (2008). Identification and characterization of a plastid-localized Arabidopsis glyoxylate reductase isoform: comparison with a cytosolic isoform and implications for cellular redox homeostasis and aldehyde detoxification. *J. Exp. Bot.* 59 2545–2554. 10.1093/jxb/ern123 18495639 PMC2423656

[B64] Srivastava S. Brychkova G. Yarmolinsky D. Soltabayeva A. Samani T. Sagi M. (2017). Aldehyde oxidase 4 plays a critical role in delaying silique senescence by catalyzing aldehyde detoxification. *Plant Physiol.* 173 1977–1997. 10.1104/pp.16.01939 28188272 PMC5373044

[B65] Sunkar R. Bartels D. Kirch H. H. (2003). Overexpression of a stress-inducible aldehyde dehydrogenase gene from *Arabidopsis thaliana* in transgenic plants improves stress tolerance. *Plant J.* 35 452–464. 10.1046/j.1365-313X.2003.01819.x 12904208

[B66] Taylor N. L. Ströhner E. Millar H. A. (2014). “Arabidopsis organelle isolation and characterization,” in *Arabidopsis Protocols, Methods in Molecular Biology* Vol. 1062 eds Sanchez-Serrano J. L. Salinas J. (New York: Springer Business Media) 551–572. 10.1007/978-1-62703-580-4-29 24057386

[B67] Timm S. Florian A. Jahnke K. Nunes-Nesi A. Fernie A. R. Bauwe A. (2011). The hydroxypyruvate-reducing system in Arabidopsis: multiple enzymes for the same end. *Plant Physiol.* 155 694–705. 10.1104/pp.110.166538 21205613 PMC3032460

[B68] Toyokura K. Watanabe K. Oiwaka A. Kusano M. Tameshige T. Tatematsu K. (2011). Succinic semialdehyde dehydrogenase is involved in the robust patterning of Arabidopsis leaves along the adaxial-abaxial axis. *Plant Cell Physiol.* 52 1340–1353. 10.1093/pcp/pcr079 21690177

[B69] Tuin L. G. Shelp B. J. (1994). *In situ* [^14^C]glutamate metabolism by developing soybean cotyledons I. Metabolic routes. *J. Plant Physiol.* 143 1–7. 10.1016/S0176-1617(11)82089-4

[B70] Weber H. Chetelat A. Reymond P. Farmer E. E. (2004). Selective and powerful stress gene expression in Arabidopsis in response to malondialdehyde. *Plant J.* 37 877–888. 10.1111/j.1365-313X.2003.02013.x 14996219

[B71] Werner A. K. Witte C.-P. (2011). The biochemistry of nitrogen mobilization: purine ring catabolism. *Trends Plant Sci.* 16 381–387. 10.1016/j.tplants.2011.03.012 21482173

[B72] Wigge B. Krömer S. Gardeström P. (1993). The redox levels and the subcellular distribution of pyridine nucleotides in illuminated barley leaf protoplasts studied by rapid fractionation. *Physiol. Plant.* 88 10–18. 10.1111/j.1399-3054.1993.tb01754.x

[B73] Yamauchi Y. Hasegawa A. Taninaka A. Mizutani M. Sugimoto Y. (2011). NADPH-dependent reductases invoved in the detoxification of reactive carbonyls in plants. *J. Biol. Chem.* 286 6999–7009. 10.1074/jbc.M110.202226 21169366 PMC3044956

[B74] Yokota A. Haga S. Kitaoka S. (1985). Purification and some properties of glyoxylate reductase (NADP^+^) and its functional location in mitochondria in *Euglena gracilis* Z. *Biochem. J.* 227 211–216. 10.1042/bj2270211 3922357 PMC1144828

[B75] Yokota A. Kitaoka S. (1979). Occurrence and operation of the glycollate-glyoxylate shuttle in mitochondria of *Euglena gracilis* Z. *Biochem. J.* 184 189–192. 10.1042/bj1840189 118746 PMC1161693

[B76] Yu L. Jiang J. Zhang C. Jiang L. Ye N. Lu Y. (2010). Glyoxylate rather than ascorbate is an efficient precursor for oxalate biosynthesis in rice. *J. Exp. Bot.* 61 1625–1634. 10.1093/jxb/erq028 20194922 PMC2914580

[B77] Zarei A. Chiu G. Z. Yu G. Trobacher C. P. Shelp B. J. (2017). Salinity-regulated regulation of genes involved in GABA metabolism and signaling. *Botany* 95 621–627. 10.1139/cjb-2016-0304

[B78] Zarei A. Körbes A. P. Younessi P. Montiel G. Champion A. Memelink J. (2011). Two GCC boxes and AP2/ERF-domain transcription factor ORA59 in jasmonate/ethylene-mediated activation of the PDF1.2 promoter in Arabidopsis. *Plant Mol. Biol.* 75 321–331. 10.1007/s11103-010-9728-y 21246258 PMC3044237

[B79] Zarei A. Trobacher C. P. Shelp B. J. (2016). Arabidopsis aldehyde dehydrogenase 10 family members confer salt tolerance through putrescine-derived 4-aminobutyrate (GABA) production. *Sci. Rep.* 6:35115 10.1038/srep35115 PMC5057122 27725774

