# Supplementary material for: Plant Glyoxylate/Succinic Semialdehyde Reductases: Comparative Biochemical Properties, Function during Chilling Stress, and Subcellular Localization
Source: Front Plant Sci. 2017 Aug 14;8:1399. doi: 10.3389/fpls.2017.01399 (PMC5558127; doi:10.3389/fpls.2017.01399)
Supplement: Supplementary file 1 [file Presentation_1.PDF]

SUPPLEMENTAL DATA FILES

Plant Glyoxylate/Succinic Semialdehyde Reductases: Comparative Biochemical Properties, Function During Chilling Stress, and Mitochondrial Localization

Adel Zarei[1], Carolyne J. Brikis[1], Vikramjit S. Bajwa[1], Greta Z. Chiu[1], Jeffrey P. Simpson[1], Jennifer R. DeEll[2], Gale G. Bozzo[1], and Barry J. Shelp[1*]

[1]Department of Plant Agriculture, University of Guelph, Guelph, Ontario, Canada. [2]Ontario Ministry of Agriculture Food and Rural Affairs, Simcoe, Ontario, Canada. [3]

SUPPLEMENTARY FIGURES

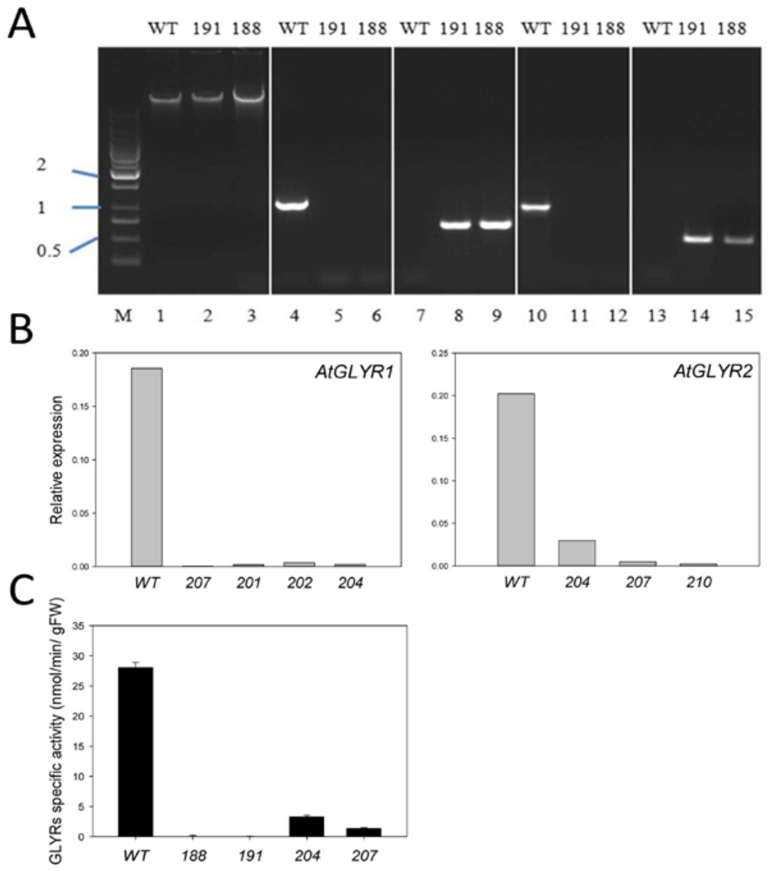

Supplementary Figure S1. Characteristics of GLYR T-DNA knockout and RNAi lines of Arabidopsis. **(A)** Molecular analysis demonstrating that selected *glyr1/glyr2* progeny are homozygous for the T-DNA insert in both *GLYR1* and *GLYR2* genes. Lanes 1-3 contain samples of genomic DNA used as templates for PCR. Lanes 4, 5, 6, and 10, 11, 12 show products of PCR reactions performed with primers specific for the WT alleles of *GLYR1* and *GLYR2*, respectively, resulting in fragments of about 1000 bp. Lanes 7, 8 and 9 show products of PCR reactions performed with primers specific for the T-DNA insert in *GLYR1*, giving a fragment of 700 bp. Similarly, lanes 13, 14 and 15 shows PCR reactions for T-DNA insertions in *GLYR2*, giving a 460 bp fragment. Numbers 191 and 188 represent different offspring selected from a cross between *glyr1* and *glyr2* T-DNA knockout lines. Lane M contains DNA marker and fragment sizes in kb are indicated at the left. (**B)** Relative expression of *GLYR1* in WT, one *glyr2* RNAi line in T-DNA *glyr1* mutant background (207) and three *glyr1* RNAi lines in T-DNA *glyr2* mutant background (201, 202 and 204). Relative expression of *GLYR2* in WT, two *glyr1* RNAi lines in T-DNA *glyr2* mutant background (203 and 204) and two *glyr2* RNAi lines in T-DNA *glyr1* background (207 and 210). Relative gene expression was measured by qPCR using rosette leaves cDNA and housekeeping gene *EF-1α*. (C) GLYR activity in cell-free extracts of the various GLYR lines selected for further experimentation.

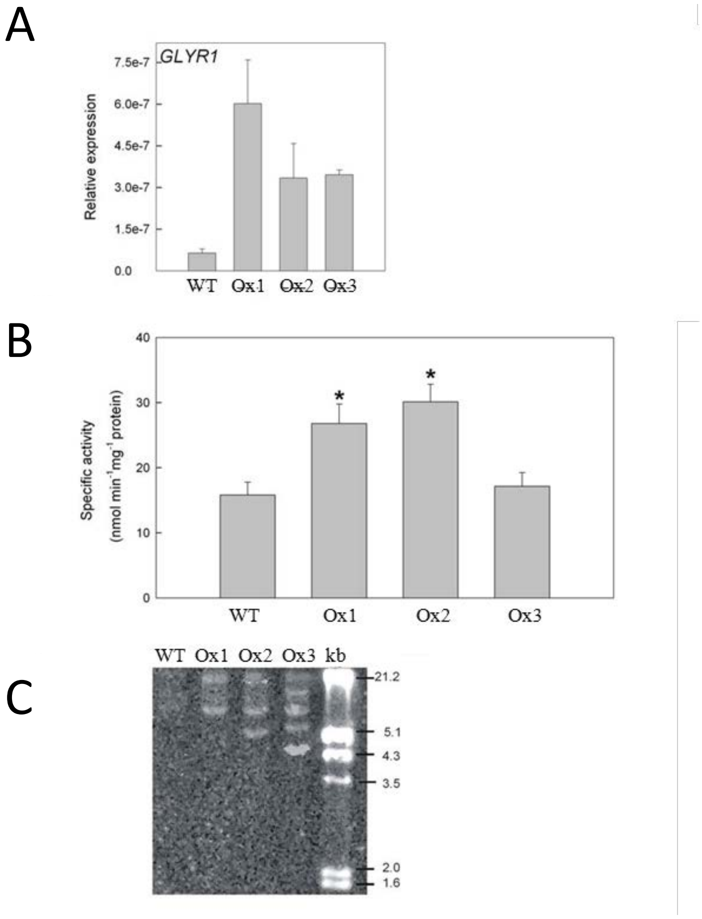

Supplementary Figure S2. Characteristics of select Arabidopsis GLYR1 overexpression (Ox) lines. (A) Expression of *GLYR1* normalized to the housekeeping gene *18S* rRNA. Data represent the mean ± SE of 2-3 biological replicates. (B) Total glyoxylate-dependent GLYR activity in desalted cell-free extracts. Data represent the mean ± SE of three biological replicates. Asterisks indicate significant difference from the WT ($P \leq 0.05$). (C) Southern blot of genomic DNA from WT and GLYR1 Ox lines. DNA was digested with *NcoI*, separated on a 1% agarose gel and transferred to a positively charged nylon membrane. The membrane was hybridized with a DIG-11-dUTP labeled 411-bp region within the *GLYR1* coding sequence.

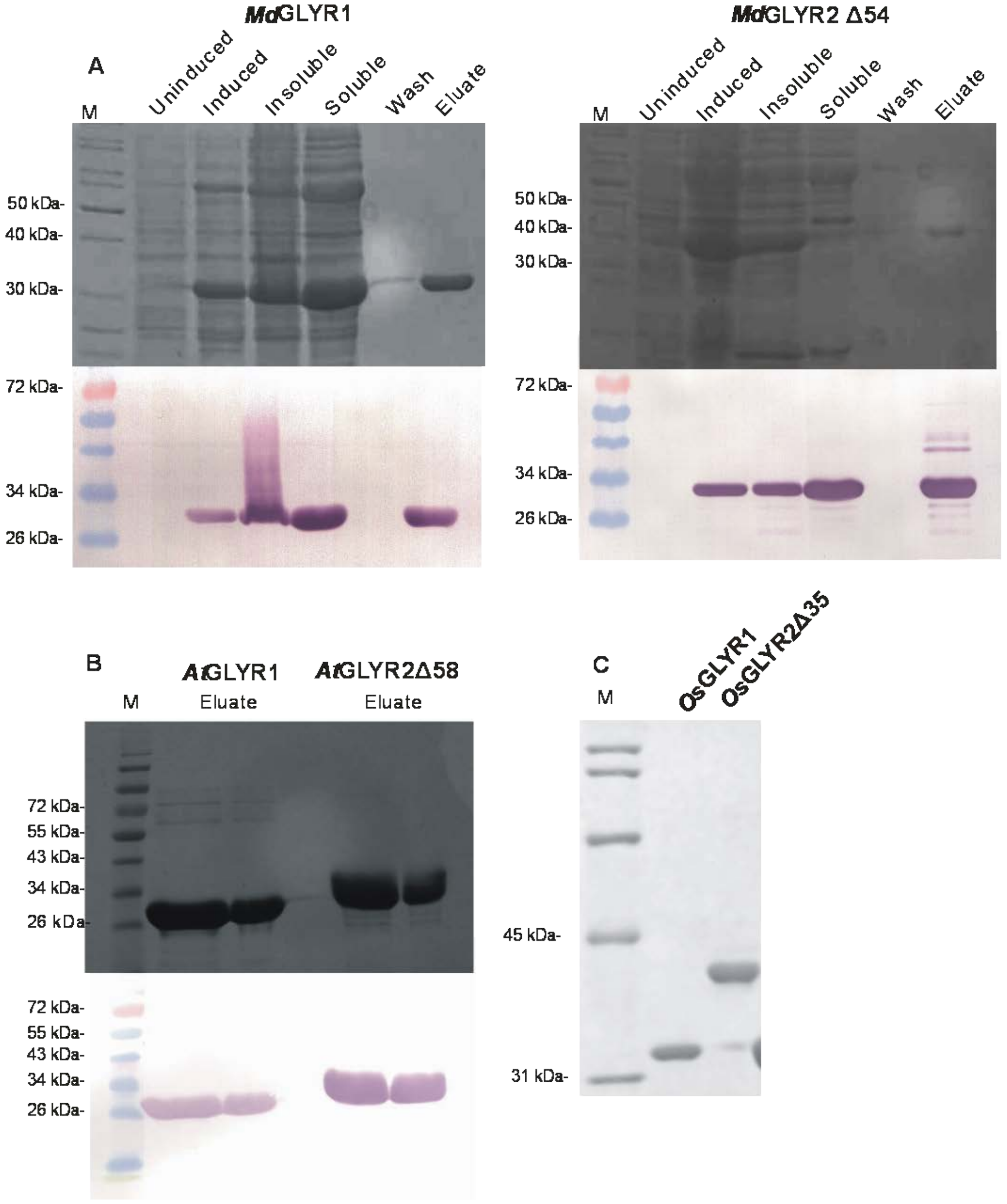

Supplementary Figure S3. Purification of recombinant apple (A), Arabidopsis (B) and rice (C) GLYRs. In A and B, top and bottom panels represent coomassie brilliant blue-stained SDS-PAGE gels and the corresponding immunoblots probed with an anti-His-tag antibody of the various fractions obtained during Ni^{2+}-affinity purification of *Md*GLYR1 and *Md*GLYR2Δ54 and *At*GLYR1 and *At*GLYR2Δ58 from *E. coli*. In C, an immunoblot probed with an anti-His-tag antibody of the eluate fraction obtained after purification of *Os*GLYR1 and *Os*GLYR2Δ*35*. Molecular weight markers (in kDa) are shown in lane M (kDa).

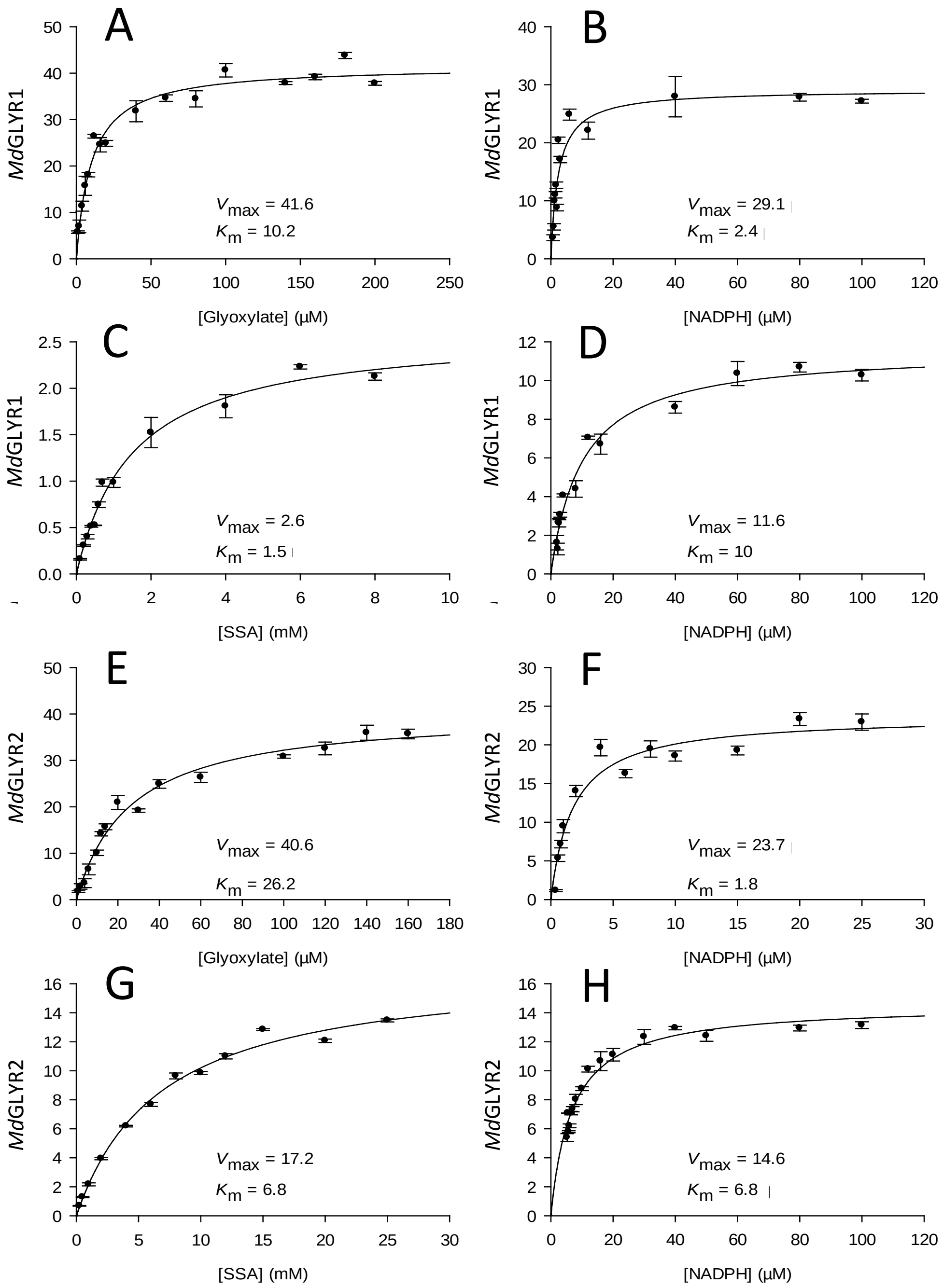

Supplementary Figure S4. Dependence of recombinant GLYR1 (A-D) and GLYR2 (E-H) specific activities (µmol min^{-1} mg^{-1} protein) from apple on glyoxylate in the presence of NADPH (A, E), NADPH in the presence of glyoxylate (B, F), SSA in the presence of NADPH (C, G), and NADPH in the presence of SSA (D, H). Data represent the mean ± SD of four technical replicates from a typical enzyme preparation.

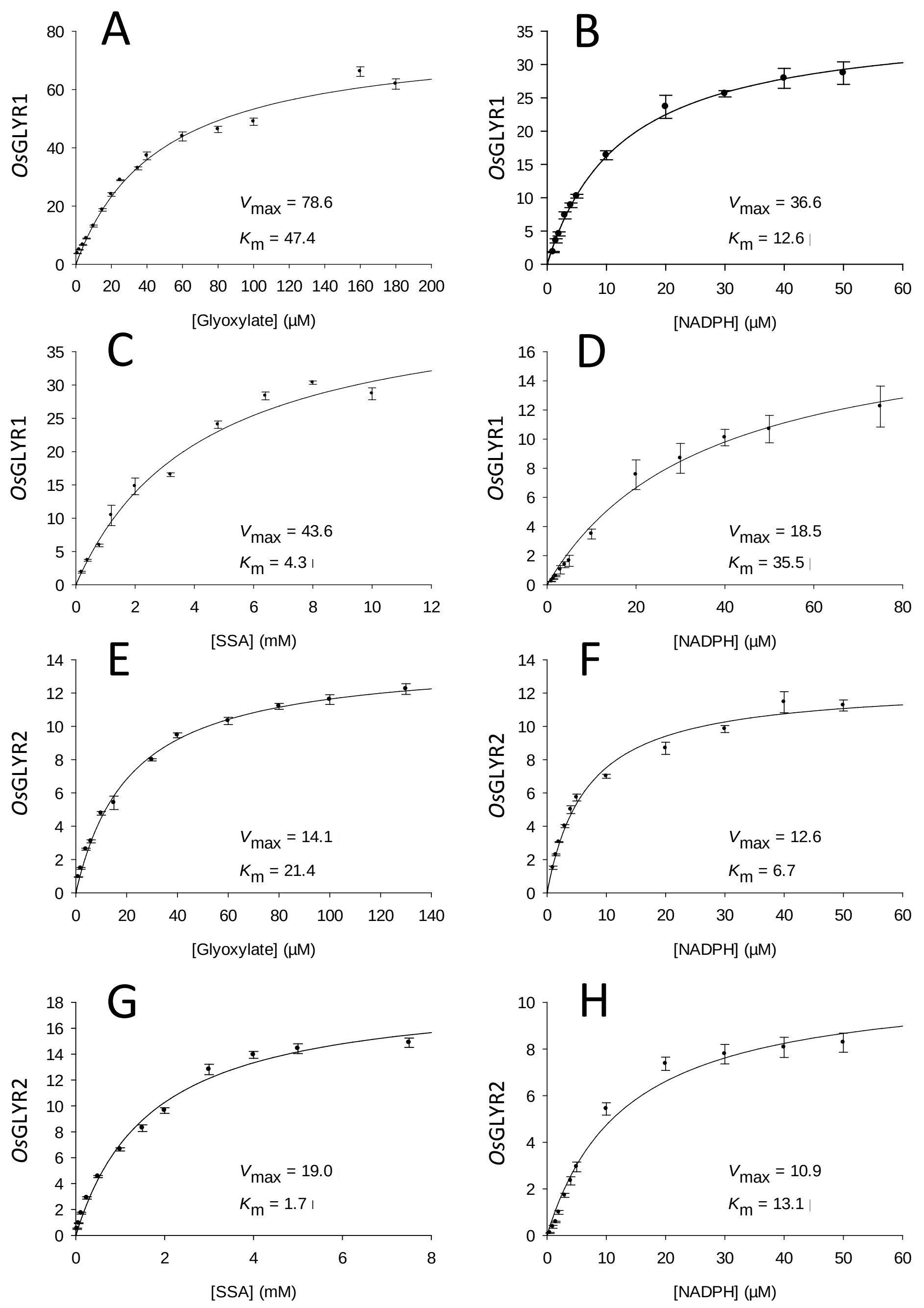

Supplementary Figure S5. Dependence of recombinant GLYR1 (A-D) and GLYR2 (E-H) specific activities (μmol min^{-1} mg^{-1} protein) from rice on glyoxylate in the presence of NADPH (A, E), NADPH in the presence of glyoxylate (B, F), SSA in the presence of NADPH (C, G), and NADPH in the presence of SSA (D, H). Data represent the mean ± SD of four technical replicates from a typical enzyme preparation.

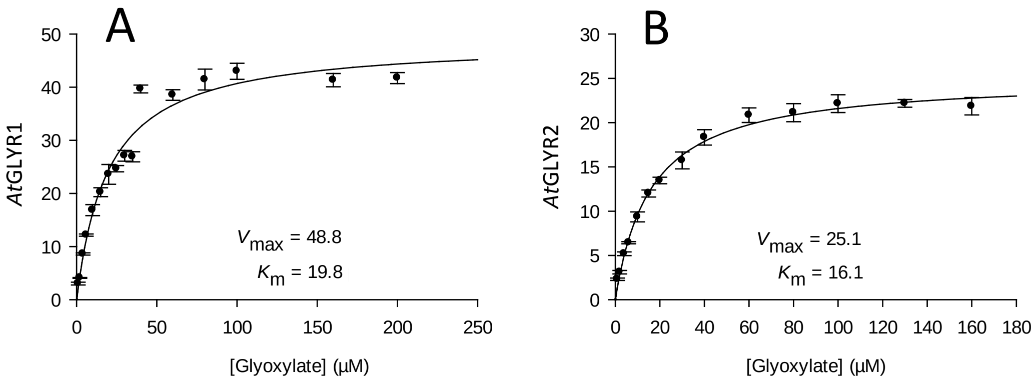

Supplementary Figure S6. Dependence of recombinant GLYR1 (A) and GLYR2 (B) specific activities (µmol min^{-1} mg^{-1} protein) from Arabidopsis on glyoxylate in the presence of NADPH. Data represent the mean ± SD of four technical replicates from a typical enzyme preparation.

SUPPLEMENTAL TABLES

Supplementary Table S1. Synthetic oligonucleotides

Primer name	Sequence (5' – 3')
CT-F12	GATTGAGAGATTGAGAGAGAGAGTG
CT-R12	CATTGCTATCTCCCCGGCG
CT-F13	TCCCACAAACTGACAGACCAGAG
CT-R13	CGGGTCAAATTACACACATTGCG
CT-F17	CCGGCATATGGCTTCATCCAAAGATGAGTTGC
CT-R17	CGCCGGATCCCTAGTGCTTCAACTTCGGTTTC
CT-F20	GGGCCATATGGAGGTCGGGTTTCTGGG
CT-R20	CGGATCCTTAACGCAGTTGGCTGTTTCG
VB-F1	GGAATTCCATATGATGGAGGTGGGGTTC
VB-R1	CGATGGATCCTCACGCCTTGCCTGAGC
VB-F2	GGAATTCCATATGATGGCGGCGATGGCGGC
VB-F3	GGAATTCCATATGTGCTCCGCCTCCTCG
VB-R2	CGCGGATCCTCACTTGCTCTGCTCCTTTGC
VB-F4	GCATCATATGTCTACCAGAGATGAACTTGGAAC
VB-R4	GCATGGATCCCTAAGCTTCTCGGGATTTTGC
RTAtGLYR1-F	GCATGGTGCATCAGTATGTG
RTAtGLYR1-R	AAAGAGCAGCACAAGGATCA
RTAtGLYR1-F	GCATGGTGCATCAGTATGTG
RTAtGLYR1-R	AAAGAGCAGCACAAGGATCA
RTEF-1-F	TGACAGGCGTTCTGGTAAGGA
RTEF-1-R	CCAGCGTCACCATTCTTCAA
LBb1.3	ATTTGCCGATTTCGGAAC
SpeI-GLYR1-F	GACTAGTCAGATAAAATGGAAGTAGGGTTTCTGGGT
SpeI-GLYR1-R	CGACGTTTTAGGGCTCTTCGAA
18S rRNA- F	TCTGGCTTGCTCTGATGATT
18S rRNA- R	TCGAAAGTTGATAGGGCAGA
glyr1-RP	AAACGATCTCTTCCCCAAGAC
glyr1-LP	ACAATCAAAACCCAAAATCCC
glyr2-RP	ATTGCTATGCTCTCTGATCCT
glyr2-LP	AAGAGCTAGCCTCATGTCTTTCT
Gabi- T-DNA	ATATTGACCATCATACTCATTGC
Clonase-GLYR1-F	GGGGACAAGTTTGTACAAAAAAGCAGGCTGGAGCAATGAC TAACCCGAT
Clonase-GLYR1-R	GGGGACCACTTTGTACAAGAAAGCTGGGTTTCACAGCTTCA ATCACAGCAG
Clonase-GLYR2-F	GGGGACAAGTTTGTACAAAAAAGCAGGCTGATGGGCAGTA ATATTCCTCAC
Clonase-GLYR2-R	GGGGACCACTTTGTACAAGAAAGCTGGGTTCGGAGTAGAT TGCGATACG
Probe-GLYR1-F	GCTGGTGACAAGGCACTCTT
Probe-GLYR1-R	TCCTAGTCCCAAGCTTCTCG
